# XCL1 expression correlates with CD8-positive T cells infiltration and PD-L1 expression in squamous cell carcinoma arising from mature cystic teratoma of the ovary

**DOI:** 10.1038/s41388-020-1237-0

**Published:** 2020-03-02

**Authors:** Ryo Tamura, Kosuke Yoshihara, Hirofumi Nakaoka, Nozomi Yachida, Manako Yamaguchi, Kazuaki Suda, Tatsuya Ishiguro, Koji Nishino, Hiroshi Ichikawa, Keiichi Homma, Akira Kikuchi, Yutaka Ueda, Yuji Takei, Hiroyuki Fujiwara, Teiichi Motoyama, Shujiro Okuda, Toshifumi Wakai, Ituro Inoue, Takayuki Enomoto

**Affiliations:** 1 Department of Obstetrics and Gynecology, Niigata University Graduate School of Medical and Dental Sciences, Niigata, 951-8510 Japan; 2 Human Genetics Laboratory, National Institute of Genetics, Mishima, 411-8540 Japan; 3 Division of Digestive and General Surgery, Niigata University Graduate School of Medical and Dental Sciences, Niigata, 951-8510 Japan; 4 Department of Pathology, Niigata Cancer Center Hospital, Niigata, 951-8133 Japan; 5 Department of Gynecology, Niigata Cancer Center Hospital, Niigata, 951-8133 Japan; 6 Department of Obstetrics and Gynecology, Osaka University School of Medicine, Suita, 565-0871 Japan; 7 Department of Obstetrics and Gynecology, Jichi Medical University, Shimotsuke, 329-0498 Japan; 8 Department of Molecular and Diagnostic Pathology, Niigata University Graduate School of Medical and Dental Sciences, Niigata, 951-8510 Japan; 9 Division of Bioinformatics, Niigata University Graduate School of Medical and Dental Sciences, Niigata, 951-8510 Japan

**Keywords:** Tumour biomarkers, Diagnostic markers, Ovarian cancer

## Abstract

Molecular characteristics of carcinoma arising from mature cystic teratoma of the ovary (MCT) remain unclear due to its rarity. We analyzed RNA-sequencing data of 2322 pan-cancer [1378 squamous cell carcinomas (SCC), 6 adenosquamous carcinomas (ASC), and 938 adenocarcinomas (AC)] including six carcinomas arising from MCT (four SCCs, one ASC, and one AC). Hierarchical clustering and principal component analysis showed that gene expression profiles of carcinomas arising from MCT were different between each histological type and that gene expression profiles of SCCs arising MCT (MCT-SCCs) was apparently similar to those of lung SCCs. By epidermis-associated pathways activity based on gene set enrichment analysis, 1030 SCCs were divided into two groups: epidermis-signature high (head and neck, esophagus, and skin) and low (cervix, lung, and MCT). In addition to pan-SCC transcriptome analysis, cytokeratin profiling based on immunohistochemistry in the independent samples of 21 MCT-SCCs clarified that MCT-SCC dominantly expressed CK18, suggesting the origin of MCT-SCC was columnar epithelium. Subsequently, we investigated differentially expressed genes in MCT-SCCs compared with different SCCs and identified *XCL1* was specifically overexpressed in MCT-SCCs. Through immunohistochemistry analysis, we identified XCL1 expression on tumor cells in 13/24 (54%) of MCT-SCCs but not in MCTs. XCL1 expression was also significantly associated with the number of tumor-infiltrating CD8-positive T cells and PD-L1 expression on tumor cells. XCL1 produced by tumor cells may induce PD1/PD-L1 interaction and dysfunction of CD8-positive T cells in tumor microenvironment. XCL1 expression may be a novel biomarker for malignant transformation of MCT into SCC and a biomarker candidate for therapeutic response to an anti-PD1/PD-L1 therapy.

## Introduction

Malignant transformation occurs in ~1–2% of mature cystic teratomas of the ovary (MCT), and the prognosis is poor at advanced stages [1]. Because of its rarity, there is no standard therapy for this disease. Malignant transformation of MCT has also been characterized by difficulty of preoperative diagnosis and histological diversity [1–3]. Squamous cell carcinoma (SCC) is the most common histological type in malignant transformation of MCT [1, 3], which is reasonable because skin epithelium is usually found in MCT [4]. Recently, Cooke et al. analyzed genomic alterations of 25 SCCs arising from MCT (MCT-SCC) by using a gene panel with 151 cancer-related genes and reported frequent alterations of *TP53*, *PIK3CA*, and *CDKN2A* that are also frequently detected in other types of SCC [5]. However, the number of targeted genes in their study was limited, and other molecular characteristics of MCT-SCC such as transcriptome, proteome, and methylation profiles remain unclear. Specific biomarkers for the early diagnosis of malignant transformation and novel therapeutic targets, which are required in clinical practice, have not yet been identified.

The Cancer Genome Atlas (TCGA) Research Network has recently performed an extensive molecular characterization of SCC derived from four different anatomical sites (lung [6], head and neck [7], cervix [8], and esophagus [9]). Moreover, TCGA studies demonstrated molecular and biological similarities across SCCs derived from different organs [10, 11]. In line with these molecular backgrounds, the clinical efficacy of anti-PD1 antibodies has been confirmed in SCC derived from lung [12], head and neck [13], cervix [14], esophagus [15], and skin tissues [16]. However, there were different immune subtypes in pan-SCCs, and the survival benefit of anti-PD1 antibodies was limited to a subset of SCC patients [17].

In this study, we investigated the molecular characteristics of carcinomas arising from MCT by performing comprehensive analyses of genomic and transcriptomic data and immunohistochemistry-based protein expression profiles. In particular, we compared gene expression profiles of MCT-SCCs with those of SCCs derived from other anatomical sites, leading to the development of novel therapeutic strategies for MCT-SCCs.

## Results

### Patients and samples

We prepared 27 carcinoma samples arising from MCT [23 SCCs, 3 adenosquamous carcinomas (ASC), and 1 adenocarcinoma (AC)]. The clinicopathological characteristics of the 27 carcinomas arising from MCT are shown in Table 1. The median onset age of all patients was 58 (33–79) years old, and 12/27 (44%) patients were diagnosed at advanced stage (stage II–IV). Consistent with previous studies [1, 18], patients at an advanced stage (stage II–IV) had a poor prognosis (Supplementary Fig. 1). Because all cases include skin epithelial tissues, we evaluated histological findings of normal epithelium close to the cancer site and divided them into three types: cancer site close to skin epithelium (7/27, 26%), cancer site close to not otherwise specified epithelium (7/27, 26%), and not available (13/27, 48%) (Supplementary Fig. 2). We could not find a sequential change from normal epithelium to carcinoma in situ in hematoxylin and eosin (H&E) slides of our samples.

**Table 1:** Clinical characteristics of 27 carcinomas arising from mature cystic teratoma of the ovary.

Sample ID	Sequencing platform	Age (year)	FIGO Stage	Histology	Classification	Epithelium adjacent to cancer site	SCC ag (ng/ml)	Tumor size (mm)	Treatment	Reccurent site	Follow up (Month)	Outcome
#1	Exome seq and RNA seq	69	IIB	SCC	Keratinizing	NOS	4.9	120	Surgery, CT	No	79	Alive
#2	Exome seq and RNA seq	53	IA	SCC	Nonkeratinizing	NOS	2	165	Surgery, CT	No	72	Alive
#3	Exome seq and RNA seq	62	IIIC	SCC	Nonkeratinizing	NA	82.2	150	Surgery, CT	Abdominal dissemination	7	Dead
#4	Exome seq and RNA seq	59	IIB	SCC	Keratinizing	NOS	1.1	126	Surgery, CT	No	14	Alive
#5	Exome seq and RNA seq	49	IIIA	ASC	Nonkeratinizing	Skin	178.2	209	Surgery, CT	Lympho node	17	Alive
#6	Target seq	48	IC	SCC	Keratinizing	NOS	1.5	108	Surgery, CT	No	92	Alive
#7	Target seq	60	IA	SCC	Keratinizing	NOS	2.1	102	Surgery, CT	No	28	Alive
#8	Target seq	63	IC	SCC	Keratinizing	Skin	13.6	136	Surgery	No	15	Alive
#9	Not done	54	IVB	SCC	Keratinizing	NA	27.3	130	Surgery, CT	Lympho node, Lung	34	Dead
#10	Not done	55	IA	SCC	Nonkeratinizing	NA	6.6	130	Surgery, CT	No	79	Dead
#11	Not done	53	IA	SCC	Nonkeratinizing	Skin	14.3	120	Surgery	Skin	62	Alive
#12	Not done	70	IIA	SCC	Nonkeratinizing	NOS	5.4	170	Surgery	Lympho node, Liver	3	Dead
#13	Not done	60	IA	SCC	Nonkeratinizing	NA	<1	90	Surgery, CT	Lung	24	Dead
#14	Not done	38	IIIC	SCC	Keratinizing	NA	12	200	Surgery, CT	Abdominal dissemination	3	Alive
#15	Not done	33	IA	SCC	Keratinizing	NA	2.4	NA	Surgery	No	64	Alive
#16	Not done	79	IIIC	SCC	Keratinizing	NA	9.9	100	Surgery	Abdominal dissemination	4	Dead
#17	Not done	79	IIIC	ASC	Keratinizing	Skin	3.5	150	Surgery, CT	No	8	Dead
#18	Not done	36	IIIC	ASC	Nonkeratinizing	NA	1.7	137	Surgery	Abdominal dissemination	2	Dead
#19	Not done	53	IA	SCC	Nonkeratinizing	NA	8.9	150	Surgery	No	115	Alive
#20	Not done	70	IIIC	SCC	Keratinizing	NA	14.2	192	Surgery	NA	3	Alive
#21	Not done	58	IIIC	SCC	Keratinizing	Skin	2252.6	153	Surgery, CT	Abdominal dissemination Pleural effusion, ascites	3	Dead
#22	Not done	53	IC	SCC	Keratinizing	NA	2.5	296	Surgery, CT	NA	6	Alive
#23	Not done	60	IC	SCC	Nonkeratinizing	NOS	1.2	88	Surgery, CT	No	156	Alive
#24	Not done	73	IA	SCC	Nonkeratinizing	Skin	3.5	150	Surgery	No	94	Alive
#25	Not done	66	IA	SCC	Keratinizing	Skin	2.5	296	Surgery	No	80	Alive
#26	Not done	46	IA	SCC	Nonkeratinizing	NA	0.9	67	Surgery	No	3	Alive
#27	RNA seq	79	IA	AC	NA	NA	9.9	100	Surgery	No	24	Alive

*SCC* squamous cell carcinoma, *ASC* adenosquamous carcinoma, *AC* adenocarcinoma, *NOS* not otherwise specified, *NA* not available, *CT* chemotherapy.

### Genomic alterations of SCC arising from MCT

We used fresh frozen samples of four MCT-SCCs and one MCT-ASC to perform whole-exome and RNA sequencing. Exome sequencing data analysis showed that *TP53* and *PIK3CA* were mutated in four out of five samples (80%) (Supplementary Table 1), and other gene mutations were observed in less than half of MCT-SCC samples. When we determined pathogenic mutations based on mutations annotated in OncoKB [19] as pathogenic (oncogenic, likely oncogenic and predicted oncogenic) or COSMIC [20] as pathogenic (FATHHMM score ≥ 0.7), 35 gene mutations were detected as “pathogenic” (Supplementary Table 2). Mutant allele expression was confirmed in 32 out of 35 pathogenic mutations by using RNA-sequencing data. Nine amplifications and two homozygous deletions were also detected, and 40% of samples harbored at least one copy number alteration (Supplementary Table 3). Moreover, we identified three in-frame fusion transcripts by using both PRADA [21] and FusionCatcher [22] (Supplementary Table 4). In particular, we found therapeutically targetable *FGFR3–TACC3* fusion in one sample.

**Fig. 1:**
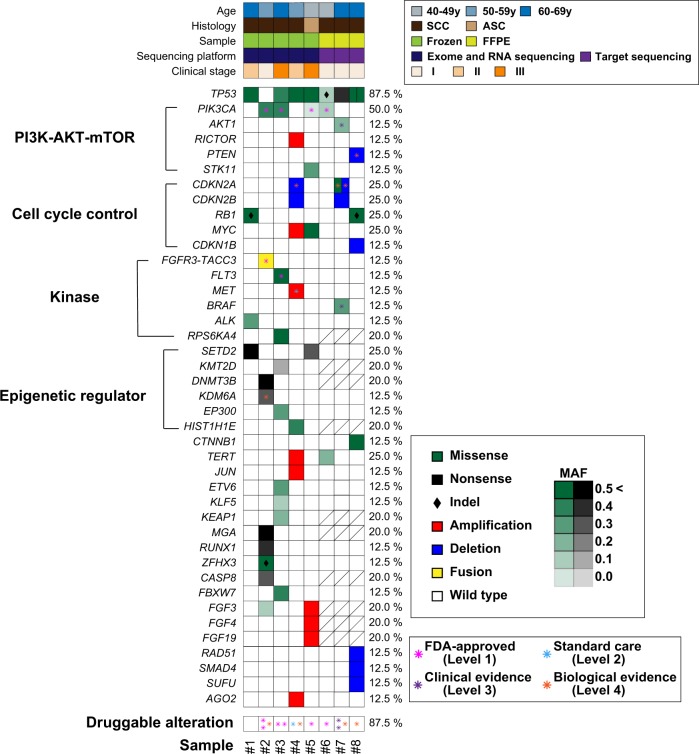
Summary of pathogenetic alterations in MCT-SCCs. Heatmaps demonstrate both sample information (upper) and distribution of genetic alterations (lower). In the lower heatmap, color and density indicate the type and MAF of each somatic mutation, respectively. Black diamonds represent indels. Asterisks indicate druggable alterations annotated in OncoKB on August 6, 2019. An oblique line in a square indicates that data are not available.

To increase the number of samples, we tried to sequence six formalin-fixed paraffin-embedded (FFPE) MCT-SCC samples using a gene panel containing 435 cancer-associated genes (CANCERPLEX®) [23]. After a quality control check, we analyzed target-gene panel data in three samples. The details of oncogenic alterations identified by the gene panel are shown in Supplementary Table 5. We used a heatmap to illustrate the summary of pathogenic alterations in a total of eight samples (Fig. 1). *TP53*, *PIK3CA*, *SETD2*, and *RB1* were frequently mutated in carcinomas arising from MCT (87.5, 50.0, 25.0, and 25.0%), and at least one well-known oncogenic alteration was identified in all samples. Overall, genes associated with the PI3K–mTOR pathway, cell cycle pathway, protein kinase, and epigenetic regulator were frequently altered, and at least one druggable oncogenic alteration was detected in seven samples (87.5%) (Supplementary Table 6). We also evaluated tumor mutation burden (TMB) that was a promising biomarker for an immune checkpoint therapy [24, 25]. The median TMB of eight samples was 4.2 mutations per megabase (range 0.8–9.9) (Supplementary Table 7).

Next, we compared the frequency of recurrent mutated genes (*TP53, PIK3CA*, *SETD2*, and *RB1*) in MCT-SCCs with that in publicly available pan-cancer data (Supplementary Fig. 3). *TP53 and PIK3CA* were also commonly mutated in SCC from other anatomical sites. However, the frequencies of *PIK3CA* (E542K and E545K mutations), the *SETD2* nonsense mutation, and the *RB1* frameshift mutation were relatively high in MCT-SCC compared with SCC or AC arising in other anatomical sites.

### Gene expression profiling of SCC arising from MCT

**Fig. 2:**
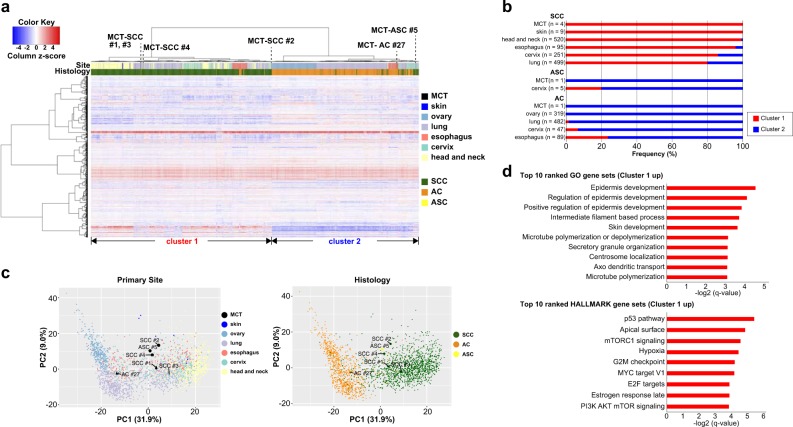
Gene expression profile of carcinoma arising from MCT compared with pan-cancer. **a** A heatmap demonstrates the hierarchical clustering using the top 1000 variable genes of all samples (*n* = 2322). The color bar and legend show the primary site and histology of the samples, respectively. **b** The distribution of clusters 1 and 2 per tumor type stratified by histological type is shown. **c** PCA using the top 1000 variable genes of all samples (*n* = 2322) is shown. The color and legend in each plot show the primary site and histology, respectively. **d** Ten top-ranked overrepresented pathways in cluster 1 based on GSEA by using HALLMARK gene sets and GO gene sets are shown.

We used RNA-sequencing data to compare gene expression profiles among six carcinomas arising from MCT (four SCCs, one ASC, and one AC), nine cutaneous SCCs, and 2307 TCGA samples (1365 SCCs, 5 ASCs, and 937 ACs). We extracted the top 1000 variable genes of all samples (*n* = 2322) to perform hierarchical clustering analysis (Fig. 2a). As a result, samples were divided into two groups that corresponded to histological types (SCC or AC). All MCT-SCCs were classified in SCC-dominant cluster 1, and one MCT-AC was classified in AC-dominant cluster 2, which is consistent with histological types of carcinoma arising from MCT. The distribution of clusters 1 and 2 per tumor type stratified by histological type is shown in Fig. 2b. Intriguingly, MCT-ASC was classified into a subcluster of cluster 2 composed of both SCC and AC samples, and four out of five cervical ASCs were also classified in the same subcluster. Principal component analysis (PCA) using the same variable 1000 genes demonstrated a clear distinction between SCC and AC (Fig. 2c). Then, we performed gene set enrichment analysis (GSEA) to identify differences in molecular characteristics between these two clusters. After multiple testing corrections using the Benjamini–Hochberg FDR method, 822 and 23 categories were significantly overrepresented in cluster 1 when we used GO gene sets and HALLMARK gene sets in GSEA, respectively. On the other hand, no category was significantly overrepresented in cluster 2 (Supplementary Data 1). Significant enrichment of epidermis-associated pathways (GO0008544, GO0045682, GO0045684, and GO0043588) was observed in SCC-dominant cluster 1 (Fig. 2d). Significant enrichments of p53 signaling, PI3K–AKT–mTOR signaling, and cell cycle signaling were also observed in SCC-dominant cluster 1, which corresponded to frequent genomic alterations in MCT-SCC.

### Similarity among pan-SCC based on integrated analyses of transcriptomic and immunohistochemistry data

Although human papilloma virus (HPV) infection is associated with the occurrence of oropharyngeal cancer and cervical cancer, there were no obvious HPV-positive cases in any MCT-SCC/ASC samples, which is in line with a previous comprehensive study [5]. Then, we focused on HPV-negative SCC because having well known that HPV-negative tumors have different biological characteristics compared with HPV-positive cancers [17, 26].

**Fig. 3:**
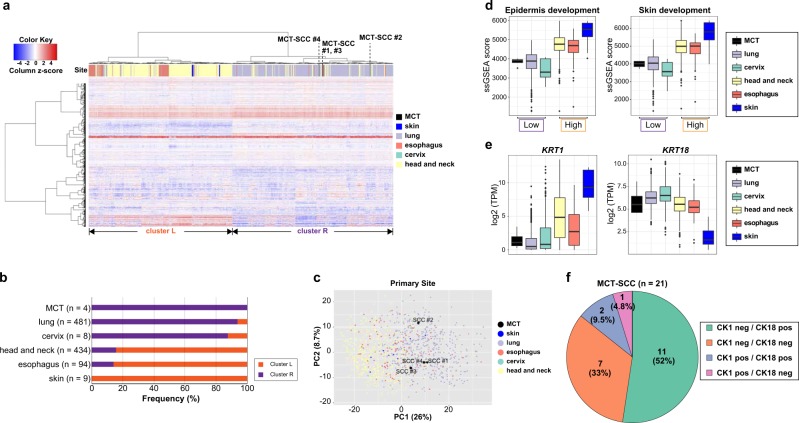
Similarity among pan-SCCs based on integrated analyses of transcriptomic and immunohistochemistry data. **a** A heatmap demonstrates the hierarchical clustering using the top 1000 variable genes in 1030 SCC patients. The color bar and legend show the primary site of the tumor. **b** The distribution of clusters L and R per tumor type stratified by histological types is shown. **c** PCA using the variable 1000 genes of 1030 SCC patients is shown. The color and legend in each plot show the primary site of the tumor. **d** Box plots show ssGSEA score of epidermis development and skin development in different SCCs. **e** Box plots show log2 TPM of *KRT1* and *KRT18* in different SCCs. **f** Pie charts show the number and percentage of CK1/CK18 protein expression evaluated by using immunohistochemistry in 21 MCT-SCCs.

We used the top 1000 variable genes in 1030 curated SCC samples to conduct hierarchical clustering and PCA plots. By hierarchical clustering analysis, SCC samples were divided into two clusters (cluster L and cluster R) mainly by primary tumor site (Fig. 3a, b). All MCT-SCCs were located in cluster R, which mainly consisted of lung SCC. On the other hand, all cutaneous SCCs were classified in cluster L, which consisted of major parts of head and neck SCCs and esophagus SCCs. A PCA plot using the same 1000 variable genes also demonstrated the distinction between head and neck and lung SCC (Fig. 3c). MCT-SCCs were located in close proximity to lung SCCs in the PCA plot. When we performed GSEA to use GO gene sets and HALLMARK gene sets, 18 and 8 categories were significantly overrepresented in cluster L, respectively (Supplementary Data 2). No category was significantly overrepresented in cluster R. Interestingly, 15 of 18 (83%) overrepresented GO categories in cluster L were common to overrepresented in cluster 1 (SCC-dominant cluster). Similarly, five of eight (63%) overrepresented categories of HALLMARK gene sets in cluster L were in common with those of cluster 1 (SCC-dominant cluster) (Supplementary Fig. 4). In particular, epidermis-associated pathways were commonly overrepresented in both cluster L and cluster 1. Then, we performed single sample GSEA (ssGSEA) to compare the pathway activity of two representative epidermis-associated pathways (GO:0008544 and GO:00043588) in different types of SCCs. Based on the above results, SCCs were divided into two epidermis-associated pathways activity subgroups: high (head and neck, esophagus, and skin) and low (cervix, lung, and MCT) (Fig. 3d, Supplementary Fig. 5).

For further characterization of MCT-SCC, we focused on cytokeratin genes composed of epidermis-associated pathways. The cytokeratin gene (*KRT1-20*) expression pattern was obviously different between SCC and AC (Supplementary Fig. 6). The expression patterns of *KRT1* and *KRT18* were clearly different between MCT-SCC and cutaneous SCC (Fig. 3e). Next, we performed immunohistochemistry on an additional 21 MCT-SCC FFPE samples by using CK1 and CK18 antibodies. CK1 and CK18 were expressed in 3/21 (14%) and 13/21 (62%) of MCT-SCCs, and the skin SCC pattern (CK1 positive and CK18 negative) was observed in only one MCT-SCC sample (Fig. 3f).

### Identification of *XCL1* overexpressed specifically in MCT-SCC

**Fig. 4:**
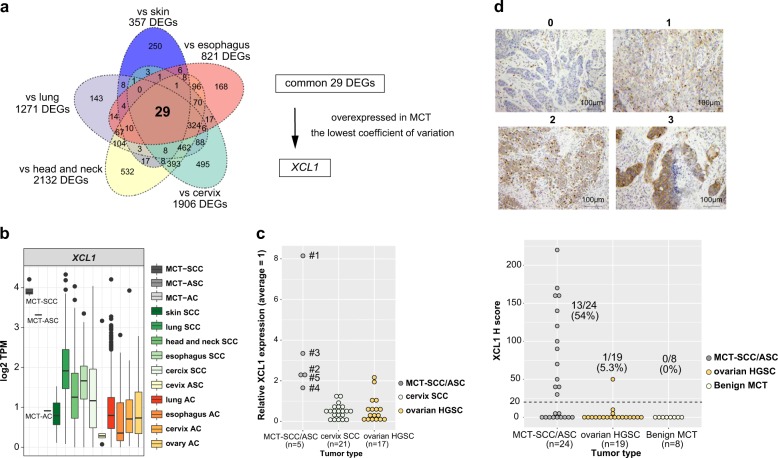
Identification of *XCL1* overexpressed specifically in MCT-SCC. **a** Venn diagram shows the number of differentially expressed genes (DEGs) between MCT-SCC and each SCC using TCC based on an FDR *q* value < 0.05. **b** Box plots show log2 TPM of *XCL1* in different types of SCCs, ASCs, and ACs. **c** Dot plots show relative *XCL1* expression among MCT-SCC/ASC (*n* = 5), cervix SCC (*n* = 21), and ovarian HGSC (*n* = 17) samples by using real-time RT-PCR. **d** Dot plots show XCL1 expression of MCT-SCC/ASC (*n* = 24), ovarian HGSC (*n* = 19), and benign MCT (*n* = 8) samples evaluated by using immunohistochemistry. The H score was obtained by the following formula: 3 × percentage of strongly stained cytoplasm + 2 × percentage of moderately stained cytoplasm + percentage of weakly stained cytoplasm, giving a range of 0–300. The dotted line indicates the lower cutoff value for a positive result (H score = 20).

We analyzed differentially expressed genes (DEGs) between MCT-SCC and each of the other SCCs arising from different anatomical sites. Compared with all types of SCCs arising from various anatomical sites, 29 genes were differentially expressed in MCT-SCC (Fig. 4a). Of these, *XCL1* was stably overexpressed in MCT-SCC compared with other SCCs. *XCL1* was overexpressed in MCT-SCC and MCT-ASC but not in MCT-AC (Fig. 4b). To validate the specificity of *XCL1* overexpression in MCT-SCC/ASC, we used 5 MCT-SCC/ASCs, 21 cervical SCCs, and 17 ovarian high-grade serous carcinoma (HGSC) samples to perform quantitative RT-PCR for *XCL1* mRNA (Fig. 4c). MCC-SCC/ASC had higher expression of *XCL1* compared with that of cervical SCC or ovarian HGSC. To confirm the protein-level expression and localization of XCL1 in tumor tissue, we added immunohistochemistry for 24 MCT-SCC/ASC and 19 HGSC samples. XCL1 staining was observed in both MCT-SCC cells and some intratumor immune cells (Supplementary Fig. 7). XCL1 expression in tumor cells was detected in 13 out of 24 MCT-SCC/ASCs (54%) and 1 out of 19 HGSCs (5.3%). No obvious positive lesion was detected in eight benign MCTs (Fig. 4d and Supplementary Fig. 8).

### Clinical significance of XCL1 overexpression in MCT-SCC

**Fig. 5:**
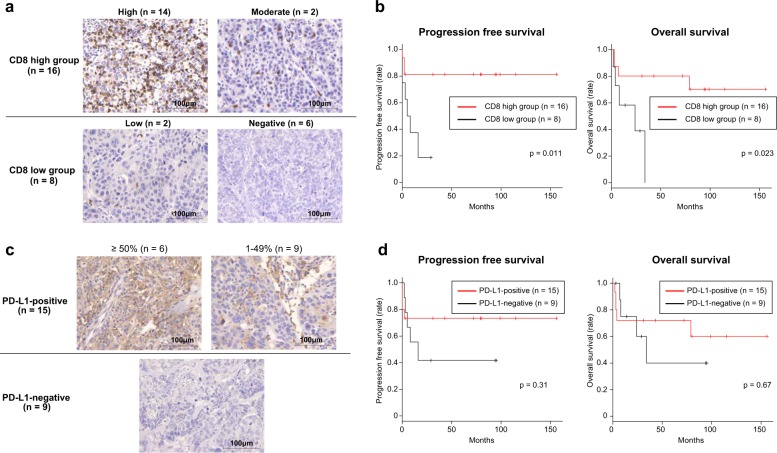
The clinical significance of the number of tumor-infiltrating CD8-positive lymphocytes and PD-L1 expression on tumor cells in 24 MCT-SCC/ASCs. **a** Immunohistochemical staining for CD8 in 24 MCT-SCC/ASCs. **b** Kaplan–Meier estimates of progression-free survival and overall survival between the CD8-high and -low groups. **c** Immunohistochemical staining for PD-L1 in 24 MCT-SCC/ASCs. **d** Kaplan–Meier estimates of progression-free survival and overall survival between PD-L1-positive and PD-L1-negative patients.

**Fig. 6:**
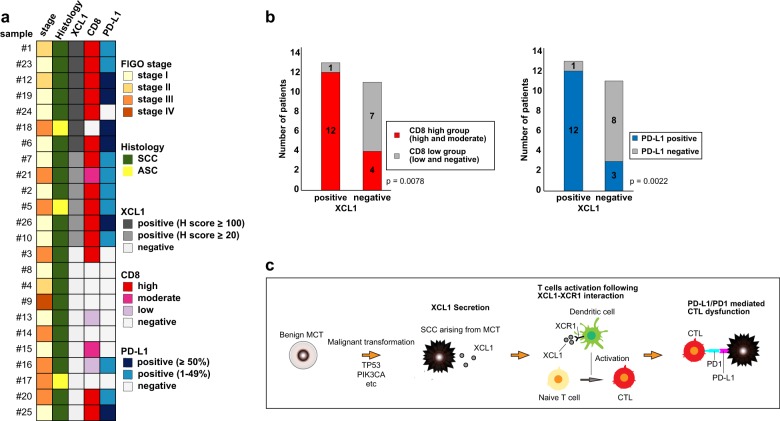
Clinical significance of XCL1 overexpression in MCT-SCC. **a** Heatmaps demonstrate both clinical information and immunohistochemical findings in 24 MCT-SCC/ASCs. **b** Bar graphs show tumor-infiltrating CD8-positive T cells and PD-L1 expression in MCT-SCC/ASCs between XCL1-positive and XCL1-neagtive patients. **c** Our hypothesis of PD-L1/PD1-mediated cytotoxic T-lymphocyte dysfunction following XCL1 production by MCT-SCC is shown.

To assess the clinical significance of XCL1 in MCT-SCC, we divided 24 MCT-SCC/ASC samples into two groups based on XCL1 expression level. Although nonkeratinizing tumors were significantly more frequent in the XCL1-high group than in the XCL1-low group (*p* = 0.019), there was no association between XCL1 expression and other clinicopathological findings in MCT-SCC/ASC (Supplementary Table 8). Moreover, there was no significant difference in progression-free or overall survival between the XCL1-high and -low groups (Supplementary Fig. 9). The XCL1–XCR1 axis plays crucial roles in the development of efficient cytotoxic immunity [27]. Therefore, we investigated the associations of XCL1 expression on tumor cells with tumor infiltration of CD8-positive T cells and PD-L1 expression on tumor cells, which is known as a biomarker for immune checkpoint inhibitors [28]. More than half of the MCT-SCC/ASCs [14/24 (58%)] showed a high number of tumor-infiltrating CD8-positive T cells (Fig. 5a), and the CD8-high group showed a significantly better prognosis than the CD8-low group (Fig. 5b). On the other hand, PD-L1 expression on more than 1% of tumor cells was observed in 15 out of 24 MCT-SCC/ASCs (63%). Of them, six samples showed PD-L1 expression on over 50% of tumor cells (Fig. 5c). There was no significant association between PD-L1 expression on tumor cells and the prognosis in MCT-SCC/ASC (Fig. 5d). We performed multivariate analysis by using XCL1, CD8, and PD-L1 expression. Of three factors, only CD8 expression was significantly associated with both progression-free survival and overall survival (Supplementary Table 9). Intriguingly, 11 of 13 (85%) XCL1-positive MCT-SCC/ASCs showed a high tumor infiltration rate of CD8-positive T cells and PD-L1 expression on tumor cells (Fig. 6a). A significant association between XCL1 expression and a high tumor infiltration rate of CD8-positive T cells was observed in MCT-SCC/ASCs (*p* = 0.0078) (Fig. 6b). In addition, there was a significant association between XCL1 expression and PD-L1 expression in MCT-SCCs (*p* = 0.0022). Subsequently, we evaluated relationships between gene expression of *XCL1* and *CD8A* or *CD274* which encodes PD-L1 in pan-SCCs and other types of SCCs by using publicly available RNA-sequencing data. No significant correlations between *XCL1* and *CD8A*/*CD274* in pan-SCCs nor any other types of SCCs were observed (Supplementary Fig. 10).

## Discussion

In this study, we demonstrated unique gene expression profiles in carcinomas arising from MCT. In particular, XCL1 was specifically overexpressed in MCT-SCC/ASC but not in SCC and AC derived from other anatomical sites and benign MCT. XCL1 expression on tumor cells was significantly associated with the number of tumor-infiltrating CD8-positive T cells and PD-L1 expression on tumor cells. Our findings suggest that XCL1 expression may be a novel biomarker for the diagnosis of malignant transformation of MCT into SCC and a biomarker candidate for therapeutic response to an anti-PD-1/PD-L1 therapy.

Consistent with a previous report [5], we detected a high frequency of *TP53* and *PIK3CA* (E542K/E545K) mutations in MCT-SCCs. Our exome sequencing analysis showed that no other genes were mutated in more than 50% of MCT-SCC. *TP53* and *PIK3CA* mutations might have a crucial role in SCC transformation from MCT [5]. In particular, most MCT-SCCs [7/8 (87.5%)] harbored at least one known oncogenic alteration in the PI3K–AKT–mTOR pathway that was targeted by many inhibitors [29]. In addition, we identified oncogenic kinase gene alterations that were druggable, and the clinical efficacy of the molecular target therapy was confirmed in various types of malignancies [19], such as *FGFR3–TACC3* fusion, *BRAF* missense mutation, *FLT3* missense mutation, and *MET* amplification. Intriguingly, the *SETD2* nonsense mutation was recurrently detected in MCT-SCC, including sample #1, in which no druggable alteration was identified. SETD2 is a histone-modifying enzyme responsible for all trimethylation of H3K36 (H3K36me3). Decreases in H3K36me3 lead to chromosomal instability, such as MMR-deficient tumors [30], and cause drug vulnerabilities [31]. Therefore, some MCT-SCC patients might obtain a new therapeutic strategy for *SETD2* mutation in the future. We evaluated TMB in eight MCT-SCC samples, and there was no obvious hypermutated tumor in our samples compared with other types of cancer [24]. Our findings suggest that integrated genomic and transcriptomic analysis could increase therapeutic drug targets in MCT-SCC.

We identified unique gene expression profiles in carcinomas arising from MCT by comparing with pan-cancer RNA-sequencing data. Three histological types of carcinomas (SCC, ASC, and AC) arising from MCT showed different gene expression patterns. Our data suggested that the expression profile of MCT-SCC was similar to that of SCCs arising from other anatomical sites. In particular, MCT-SCC showed a more similar expression pattern to lung SCC than cutaneous SCC. The origin of MCT-SCC has been discussed for decades, and there are two possible origins: epidermis [4, 32] and columnar epithelium [33, 34]. The epidermis origin theory is the most likely candidate because skin epithelium is generally found in MCT. Moreover, carcinoma in situ with a Bowen’s disease-like pattern that is typical in cutaneous SCC is found in some cases of MCT-SCCs [4, 32]. On the other hand, the columnar epithelium origin theory has also been considered because some MCT-SCCs exhibited a sequential change from respiratory epithelium to carcinoma in situ [33]. CK18-positive and CK10-negative expression patterns that were observed in lung and cervix SCCs were found in the majority of MCT-SCCs [34]. Although we could not find a sequential change from columnar epithelium to carcinoma in situ in our H&E slides of MCT-SCC samples, our results of expression data analysis support the columnar epithelium origin theory in MCT-SCC.

Although the gene expression profile of MCT-SCC was similar to that of lung SCC, we identified that XCL1 was specifically overexpressed in MCT-SCC compared with other SCCs and benign MCT. There were no significant differences in prognosis or clinical stage between the high- and low-XCL1 groups in MCT-SCCs. These results suggest that XCL1 may be a biomarker for SCC transformation from MCT but not a prognostic factor. XCL1 is a C class chemokine and is produced mainly by natural killer and activated CD8-positive T cells [35, 36]. The selective receptor XCR1 is expressed by a subpopulation of dendritic cells, and the XCL1–XCR1 axis plays a crucial role in the development of efficient cytotoxic immunity [27]. In addition, the XCL1–XCR1 interaction is involved in tumor migration, invasion, and proliferation in several malignancies [37, 38]. In MCT-SCC, we found XCL1 expression on tumor cells as well as some intratumor immune cells in XCL1-positive MCT-SCCs/ASCs. These intratumor immune cells with XCL1 expression were rarely observed in HGSCs. These findings might reflect that CD8-positive T cells activated by the XCL1–XCR1 interaction produced XCL1 [27].

Immunohistochemistry analysis demonstrated that XCL1 expression on tumor cells was positively correlated with the number of tumor-infiltrating CD8-positive T cells and PD-L1 expression on tumor cells, which were predictive biomarker candidates in an immune checkpoint therapy in various types of malignancies. Based on tumor PD-L1 expression and the presence of tumor-infiltrating lymphocytes, four different types of tumor immune microenvironment were proposed to predict the response to immune checkpoint therapies [28]. More than half of MCT-SCCs [13/24, (54%)] showed high tumor infiltration of CD8-positive T cells and high PD-L1 expression, and these cases might be treated effectively by the PD1/PD-L1 blockage therapy. XCL1 expression may be a biomarker candidate to select an appropriate therapy for MCT-SCC patients.

On the other hand, 11/24 (46%) MCT-SCC patients with low or negative tumor-infiltrating CD8-positive T cells or low PD-L1 expression tended to have poor prognosis. For these patients, other strategies such as combination therapy that is designed to bring T cells into tumors and avoid them being turned off, such as the combination of anti-CTLA-4 and anti-PD-1, might be needed [28].

No significant correlations between *XCL1* expression and CD8A/CD274 expression in pan-SCC data nor any types of SCCs were observed. Although we could not investigate the association of XCL1 expression with CD8-positive T cells infiltration/PD-L1 expression in the protein level in other SCCs, these associations might be specific to MCT-SCC. Moreover, the number of XCL1-high samples comparable to MCT-SCC in other SCCs was quite limited. Further analysis should be performed to reveal whether the association is also observed in other types of SCCs.

Our hypothesis of the tumor immune microenvironment in MCT-SCC is summarized in Fig. 6c. However, there are several important limitations in this study. Because of the tumor rarity, our sample size was small. In addition, we were not able to obtain any resources of MCT-SCC, such as the cancer cell line, for the purpose of performing functional analysis, especially the XCL1–XCR1 interaction in MCT-SCC. Further studies, including large-scale genomic and transcriptomic analyses and in vitro/vivo experiments, are required to prove our hypothesis.

In conclusion, our comprehensive genomic and transcriptomic analysis clarified the molecular characteristics of carcinomas arising from MCT. XCL1 expression might be a promising biomarker for malignant transformation of MCT into SCC and a biomarker candidate of therapeutic response to an anti-PD1/PD-L1 therapy.

## Materials and methods

### Clinical samples

This study was performed in accordance with the Declaration of Helsinki and was approved by the institutional ethics review board at Niigata University, Osaka University, Jichi Medical University, Niigata Cancer Center, and National Institute of Genetics. A total of 27 patients histologically diagnosed with carcinoma arising from MCT of the ovary between 1999 and 2018 at the Niigata University, Osaka University, Jichi Medical University, and Niigata Cancer Center were enrolled. Fresh frozen tumor tissues and FFPE samples obtained by surgical resection were collected. In addition, fresh frozen tumor tissues and FFPE samples from patients who were diagnosed with high-grade serous ovarian cancer, cervical cancer, and MCT of the ovary at Niigata University in the same period were also collected as controls. All patients provided informed consent for the collection of samples and subsequent analysis.

### Whole-exome sequencing and analysis

Before sequencing analysis, we confirmed that all fresh frozen samples histologically contained over 50% of tumor cells (Supplementary Fig. 11). Two fresh frozen samples (samples #15 and #21) were excluded because of low tumor cell content. We extracted DNA from tumor tissues and blood samples as previously described [39]. We performed whole-exome sequencing using genomic DNA derived from the five carcinomas arising from MCT samples [one sample with matched blood sample (sample #1) and four samples (sample #2, #3, #4, and #5) without matched blood samples], and detected putative somatic mutations and copy number alterations. The details of experimental methods for whole-exome sequencing and computational analyses have been previously described [39, 40].

### Detection of putative somatic mutations and copy number alterations

Detection of putative somatic mutations and copy number alterations in sample #1 was conducted as previously described [39]. For samples without matched blood samples (samples #2, #3, #4, and #5), the detection of putative somatic mutations and copy number alterations was also performed as previously described [40]. We focused on mutations and copy number alterations annotated in OncoKB [19] as pathogenic (oncogenic, likely oncogenic, and predicted oncogenic) at August 6, 2019. We also focused on mutations that were annotated in COSMIC [20], release v87 as pathogenic (FATHHMM score ≥ 0.7). We defined a threshold of ≥6 copies as gene amplification. Homozygous deletion was defined as a copy number equal to zero. To validate homozygous deletions, we examined the mRNA expression of homozygously deleted genes by using RNA-sequencing data. We excluded homozygously deleted genes showing obvious mRNA expression despite copy number 0 from copy number alteration analysis.

### Tumor mutation burden (TMB)

The TMB, defined as the rate of nonsynonymous single nucleotide variants (SNVs) per megabase, was determined for samples analyzed by using whole-exome sequencing. To estimate the TMB, only SNVs that covered at least ten reads in coding area were evaluated.

### Target sequencing analysis (CANCERPLEX®)

Six FFPE MCT-SCC samples (samples #6, #7, #8, #23, #24, and #25) obtained by surgical resection at the Niigata Cancer Center were analyzed by a gene panel containing 435 cancer-associated genes (CANCERPLEX®). The details of the 435 genes are shown in https://kewinc.com/wp-content/uploads/2017/07/Full-gene-list.pdf. An independent pathologist evaluated tumor content, and over 20% of tumor content was confirmed in all samples. DNA was extracted using the BioStic FFPE Tissue DNA Isolation Kit (MO BIO Laboratories, Carlsbad, CA, USA). Three FFPE samples (samples #23, #24, and #25) were excluded because of poor DNA quality. All sample preparation and genomic sequencing and analysis were performed in a CLIA/CAP-accredited laboratory (KEW, Cambridge, MA, USA). The details of experimental methods for next-generation sequencing and computational analyses are the same as previously described [41].

### Pan-cancer data analysis

Mutation data of TCGA samples were downloaded from TCGA Pan Cancer Atlas Studies. Mutation data of cutaneous SCC were downloaded from MSK-IMPACT using cBioPortal (http://www.cbioportal.org) [42]. The HPV status of TCGA SCC samples was downloaded from a previous study [11].

### RNA sequencing

Total RNA was extracted from frozen samples using TRIzol (Invitrogen, Carlsbad, CA, USA).

Total RNA was used for the library preparation, which was conducted using a TruSeq Stranded mRNA Library Prep Kit (Illumina, San Diego, CA, USA) according to the manufacturer’s protocol. The samples were sequenced on the Illumina HiSeq 2500 platform with the 2 × 100-bp paired-end read module. The details of the procedure were the same as previously described [40].

### Detection of fusion transcripts

To identify fusion transcripts, we used both PRADA [21, 40] and FusionCatcher [22] as previously described.

### RNA-sequencing data analysis

We downloaded RNA-sequencing data of cutaneous SCC (SRP078314) from Gene Expression Omnibus [43] and used kallisto (version 0.43.0) to perform TPM normalization of our RNA sequencing data from carcinoma arising from MCT samples as well as cutaneous SCC RNA sequencing data [44]. Gene expression data of TCGA pan-cancer samples calculated by kallisto (version 0.43.0) were downloaded from the Google Cloud Pilot RNA sequencing for CCLE and TCGA (Tatlow, PJ. 2016. “Google Cloud Pilot RNA-Sequencing for CCLE and TCGA”. OSF. July 19. osf.io/gqrz9.). To obtain one expression value per gene and sample, transcripts measuring the same gene were averaged. Then, we calculated the median absolute deviation to exclude variable genes across all samples. We performed clustering analysis of both PCA and generated a heatmap using the log10 transformed TPM value of the top 1000 variable genes across all samples. The hierarchical clustering analysis was performed by applying the absolute Pearson distance measure and Ward D algorithm. The calculated distance was plotted using the heatmap.3R package (https://www.rdocumentation.org/packages/GMD/versions/0.3.3/topics/heatmap.3). PCA was performed using the “prcomp” package in R version 3.5.2. We evaluated the validity of cluster number using both the Calinski and Harabasz index [45] and the Kzanwski and Lai index [46] (Supplementary Fig. 12). We performed GSEA using HALLMARK gene sets and GO gene sets that were downloaded from the Molecular Signatures Database (http://www.broad.mit.edu/gsea/.msigdb/msigdb_index.html). By using a normalized enrichment score and *q* value, we extracted overrepresented pathways for which the −log2 (*q* value) was >2.0. We ran ssGSEA using GO gene sets for each sample. We used the TCC R package [47] to detect DEGs (*q* value < 0.05) between MCT-SCC and each group of SCC arising from other anatomical sites. To identify important DEGs, we calculated the coefficient of variation (the ratio of the standard deviation to the mean).

### Quantitative real-time RT-PCR

We performed quantitative real-time RT-PCR as previously reported [40]. All primers used are shown in Supplementary Table 10.

### HPV genotyping

For five frozen samples (samples #1, #2, #3, #4, and #5), HPV-DNA testing targeting 16 high- and low-risk HPV genotypes (genotypes 6, 11, 16, 18, 30, 31, 33, 35, 39, 45, 51, 52, 56, 58, 59, and 66) was performed using the multiplex PCR method (PapiPlex) at the GLab Pathology Center Co., Ltd (Sapporo, Japan) [48]. In addition, for three FFPE samples (sample #6, #7, and #8), HPV-16 and HPV-18 viral sequences were analyzed by using CANCERPLEX® as previously described [23].

### Histological analysis

All histological specimens were reviewed by two gynecological pathologists (TM and TE). In immunohistochemical analysis, we excluded two stage IA MCT-SCC samples (sample #11 and sample #22) that did not contain enough tumor cells. Antibodies were purchased from the indicated suppliers: CD8 (M7103, Dako; dilution ratio 1:100), CK1 (sc-376224, Santa Cruz; dilution ratio 1:50), CK18 (ab668, Abcam; dilution ratio 1:100), PD-L1 (ab205921, Abcam; dilution ratio 1:500), and XCL1 (HPA057725, Atlas Antibodies; dilution ratio 1:200). We experimented with immunohistochemical staining as previously reported [40]. CK1 and CK18 were defined as “positive” when >10% of the tumor cells showed cytoplasmic immunoreactivity [34]. Tumor-infiltrating CD8-positive T cells were manually counted in three random high-power fields. Tumor-infiltrating T cells were graded as negative, low, moderate, or high (0, ≤5, 6–19, or ≥20 T cells per high-power field, respectively) [49]. Over 1% of PD-L1 expression on tumor cells was defined as “positive” based on the previous assessment [50]. Cytoplastic staining of XCL1 on tumor cells was evaluated on a scale of 0 (no staining) to 3 (strong staining). Subsequently, the H score was obtained by the formula: 3 × percentage of strongly stained cytoplasm + 2 × percentage of moderately stained cytoplasm + percentage of weakly stained cytoplasm, giving a range of 0–300 [51]. When the H score was higher than 20, XCL1 was defined as “positive”.

### Statistical analysis

All computations were conducted using R (R Core Team (2018): a language and environment for statistical computing. R Foundation for Statistical Computing, Vienna, Austria, http://www.R-project.org/). Fisher’s exact test was used to evaluate the significance between data groups. Spearman’s rank test was used to evaluate correlation between data groups. *P* values < 0.05 indicated statistical significance. Standard statistical tests were used as appropriate, including unpaired *t*-test and log rank test. Cox proportional model was used for the multivariate analysis.

## Supplementary information

Supplementary Information

Supplementary Data 1

Supplementary Data 2
