# Supplementary material for: XCL1 expression correlates with CD8-positive T cells infiltration and PD-L1 expression in squamous cell carcinoma arising from mature cystic teratoma of the ovary
Source: Oncogene. 2020 Mar 2;39(17):3541–54. doi: 10.1038/s41388-020-1237-0 (PMC7176584; doi:10.1038/s41388-020-1237-0)
Supplement: Supplementary file 1 — Supplementary Information [file 41388_2020_1237_MOESM1_ESM.pdf]

Supplementary Information

XCL1 expression correlates with CD8 positive T cells infiltration and PD-L1 expression in squamous cell carcinoma arising from mature cystic teratoma of the ovary

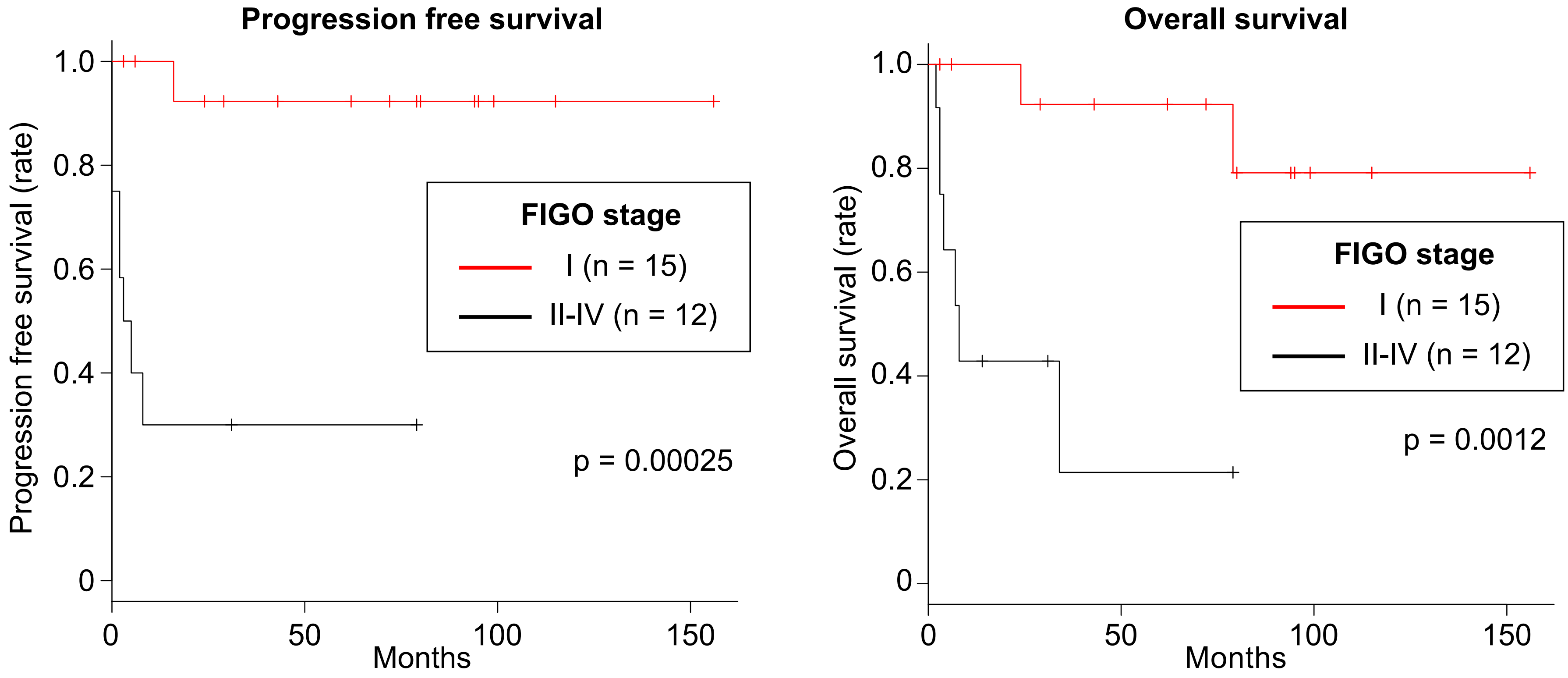

Supplementary Figure 1. The prognostic value of clinical stage in 27 carcinomas arising from MCT

Kaplan-Meier estimates of progression-free survival and overall survival for stage I and stage II-IV patients.

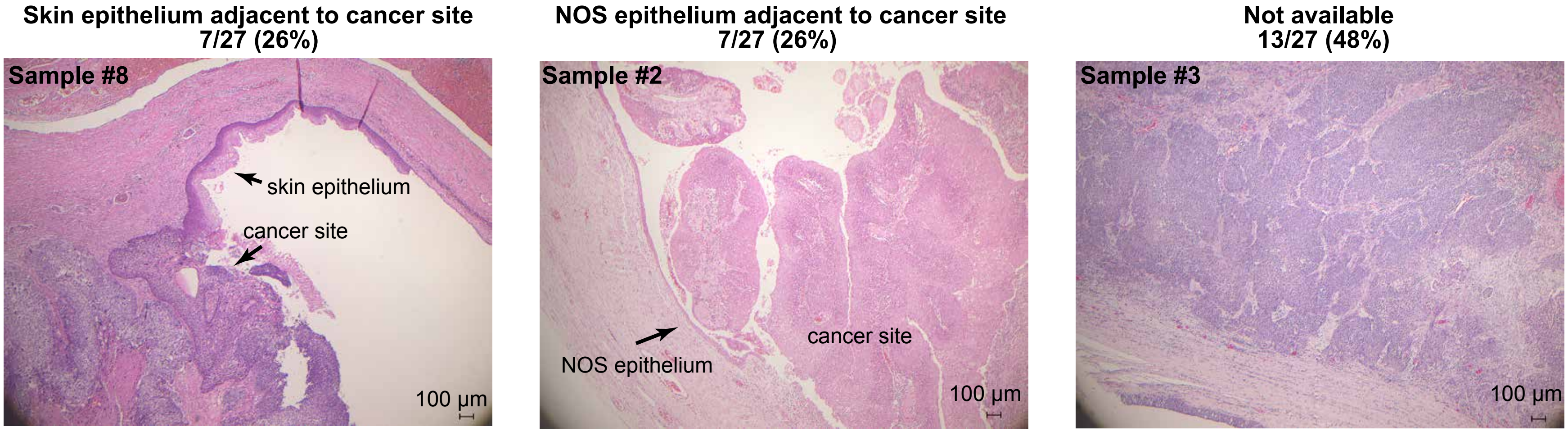

Supplementary Figure 2. Histological findings of normal epithelium close to the cancer site in 27 patients of carcinoma arising from MCT

Three types of representative images of H&E staining in 27 carcinomas arising from MCT are shown (100X magnification).

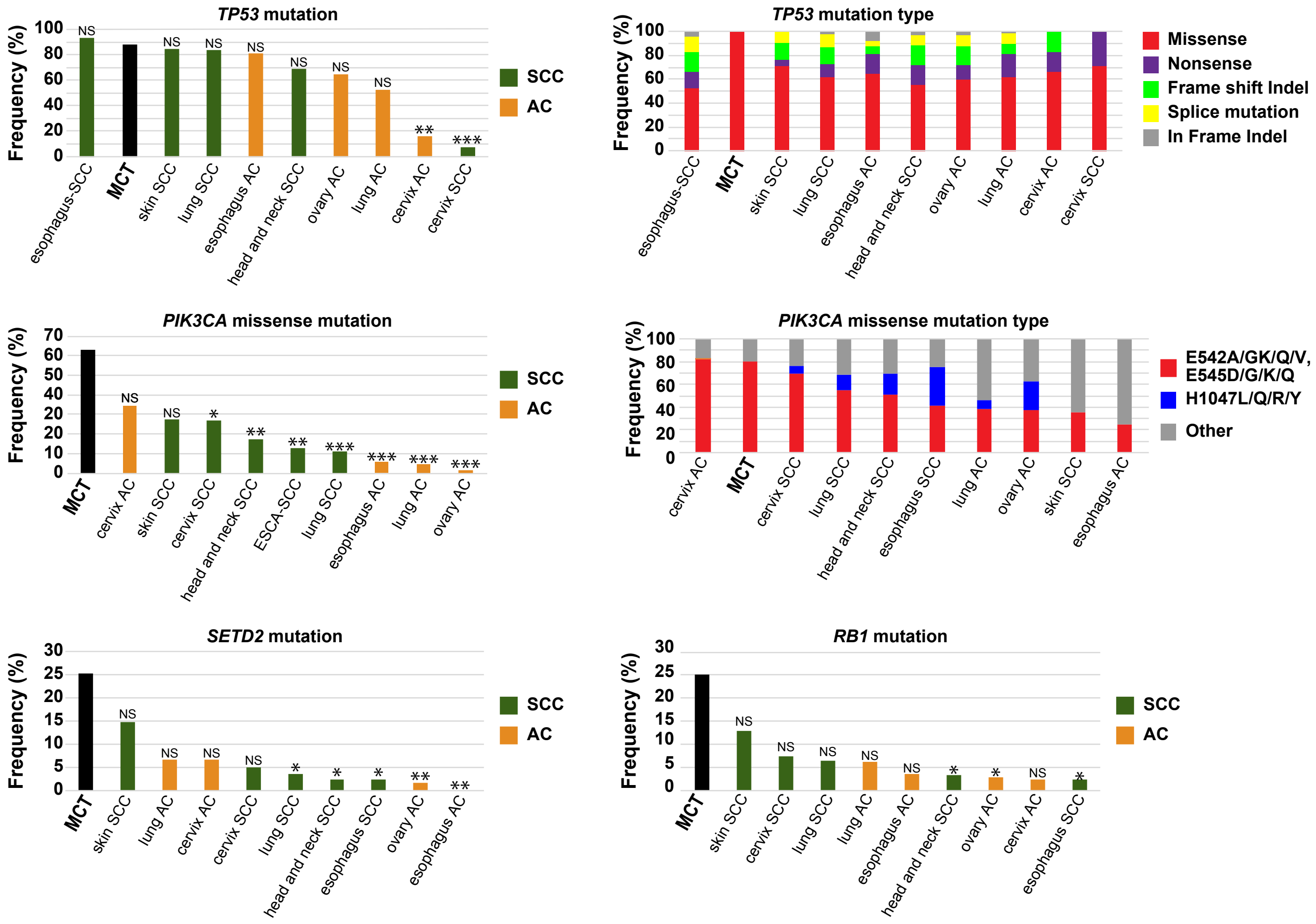

Cancer type	Number
MCT	8 (7 SCC and 1 ASC)
skin SCC	55
head and neck SCC	523
lung SCC	487
cervix SCC	251
esophagus SCC	95
ovary AC	585
lung AC	566
cervix AC	46
esophagus AC	87

Supplementary Figure 3. Comparison of the frequency of recurrent mutated genes (*TP53*, *PIK3CA*, *SETD2* and *RB1*) in MCT-SCCs with that in publicly available pan-cancer data

Bar graphs show the frequency of mutations in each tumor type. Details of mutation types in *TP53* and *PIK3CA* are also shown. NS denotes Not Significant, * denotes $p < 0.05$, ** denotes $p < 0.01$ and *** denotes $p < 0.001$, respectively (MCT vs each cancer type).

GO gene sets

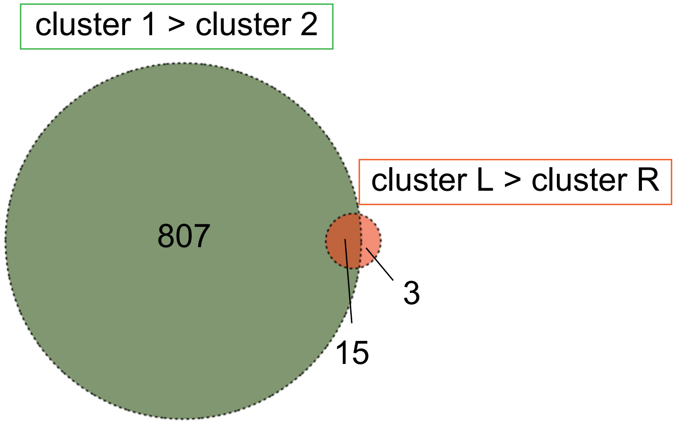

Common enrichment pathway in cluster 1 and cluster L (GO terms)
Epidermis development
Keratinocyte differentiation
Skin developement
Epidermal cell differentiation
Intermediate filament based process
Positive regulation of epidermis development
Desmosome
Intermediate filament organization
Regulation of water loss via skin
Cell substrate junction assembly
Response to corticosterone
Connexon complex
Peptide cross linking
Gap junction
Keratinization

HALLMARK gene sets

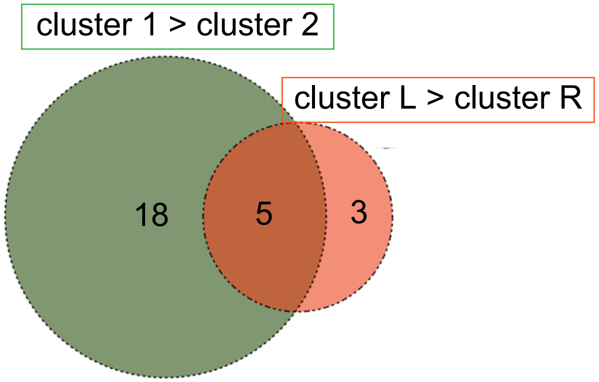

Common enrichment pathway in cluster 1 and cluster L (HALLMARK terms)
p53 pathway
Estrogen response late
Apical junction
TNFa signaling via NFKB
Estrogen response early

Supplementary Figure 4. Common enrichment pathways between cluster 1 and cluster L

Venn diagrams show the number of common enrichment pathways in GO gene sets and HALLMARK gene sets between cluster 1 and cluster L.

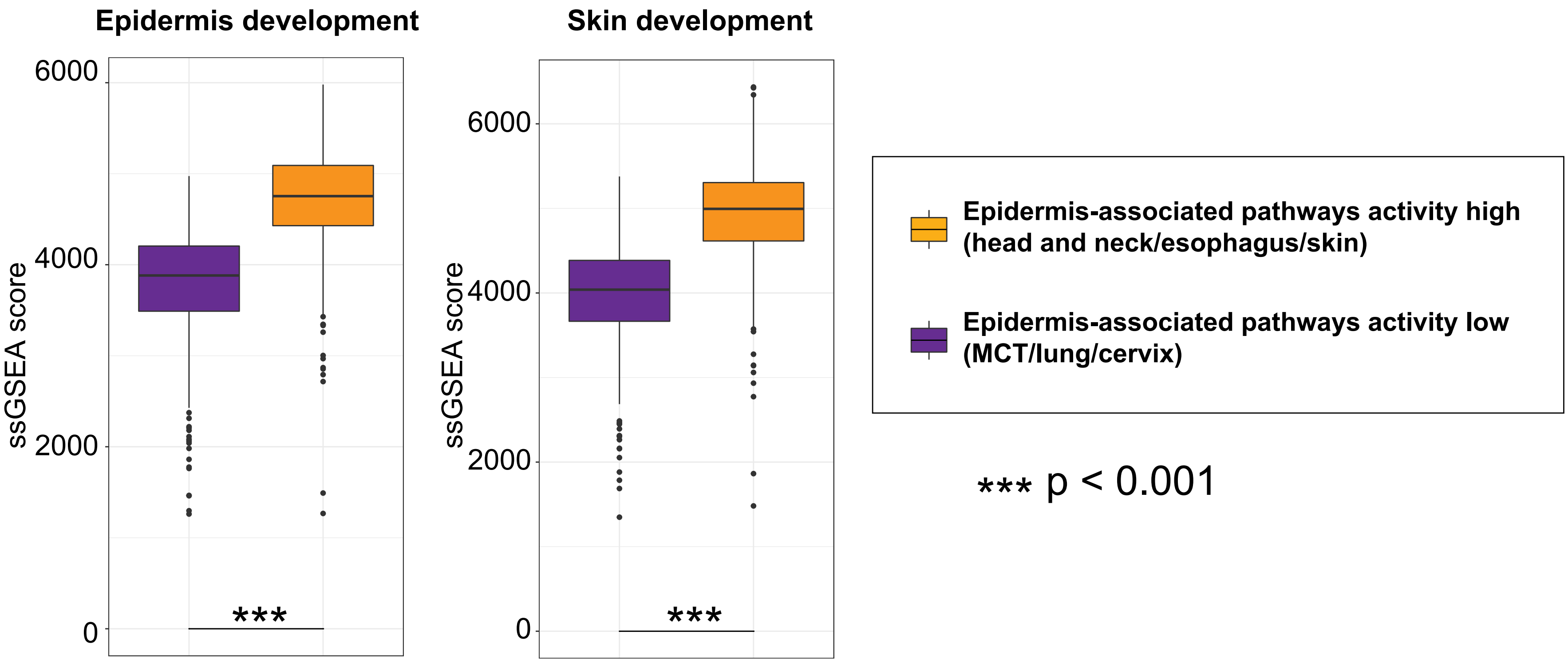

Supplementary Figure 5. Comparison of two subgroups, high (head and neck, esophagus, and skin) and low (cervix, lung, and MCT) epidermis-associated pathways activity subgroups

Boxplots show ssGSEA score of Epidermis development and Skin development in high (head and neck, esophagus and skin) and low (MCT, lung and cervix) epidermis-associated pathways activity subgroups. (*** denotes $p < 0.001$).

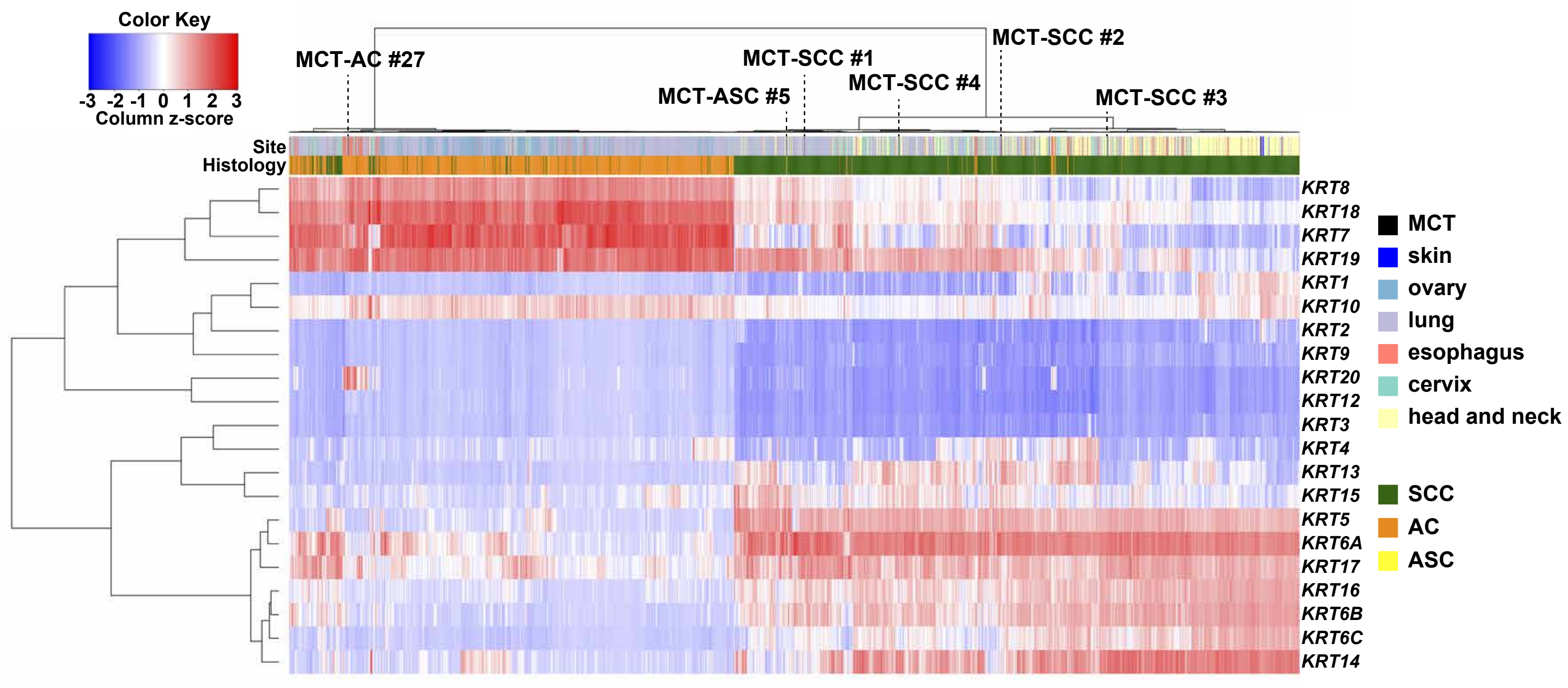

Supplementary Figure 6. KRT gene expression in pan-SCCs and pan-ACs

A heatmap demonstrates the hierarchical clustering of the 21 KRT genes from 2,322 patients. The color bar and legend show the primary site and histology of the samples, respectively.

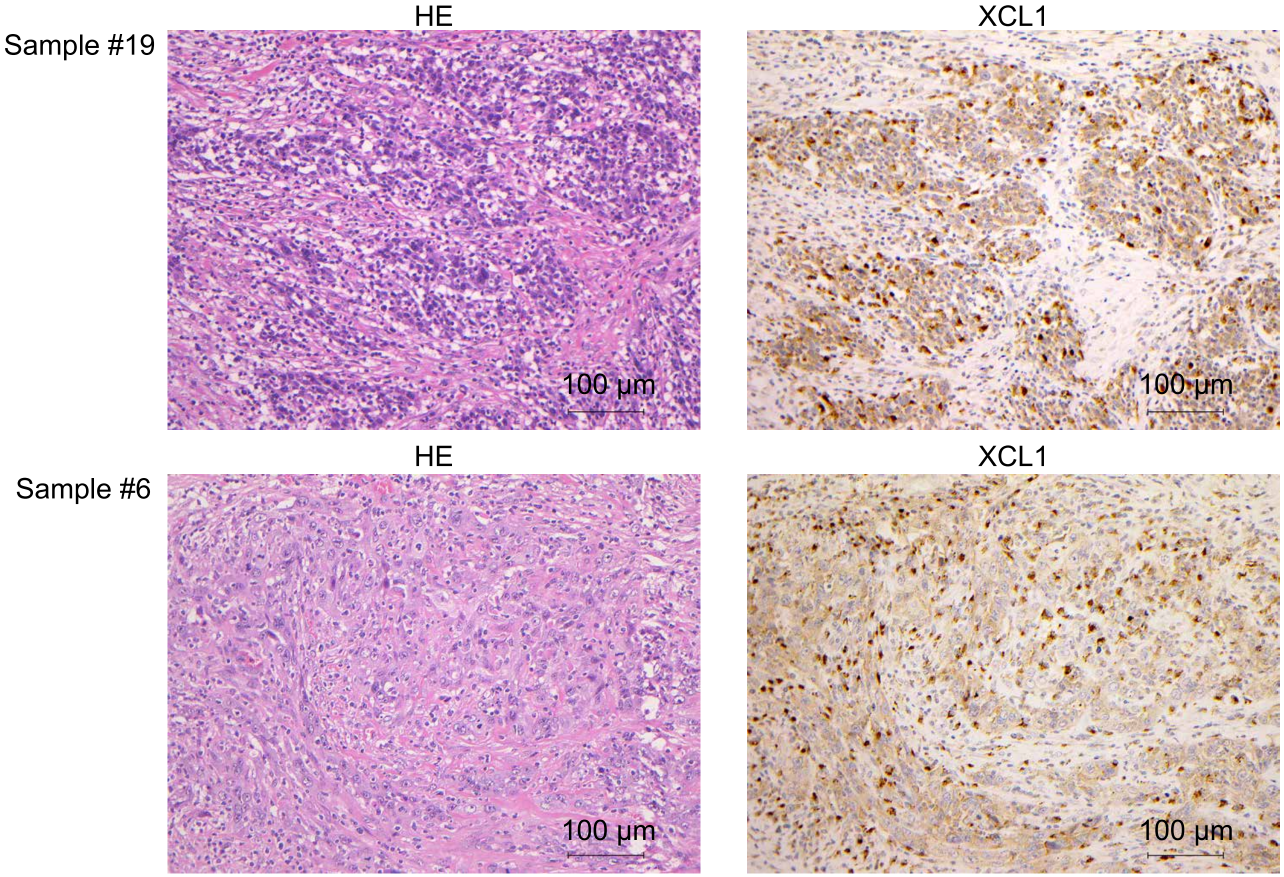

Supplementary Figure 7. Histological findings of sample #19 and sample #6

Representative images of H&E staining and immunohistochemical staining for XCL1 in samples #19 and #6 are shown (200X magnification). XCL1 staining was observed in both MCT-SCC cells and some intratumor immune cells (200X magnification).

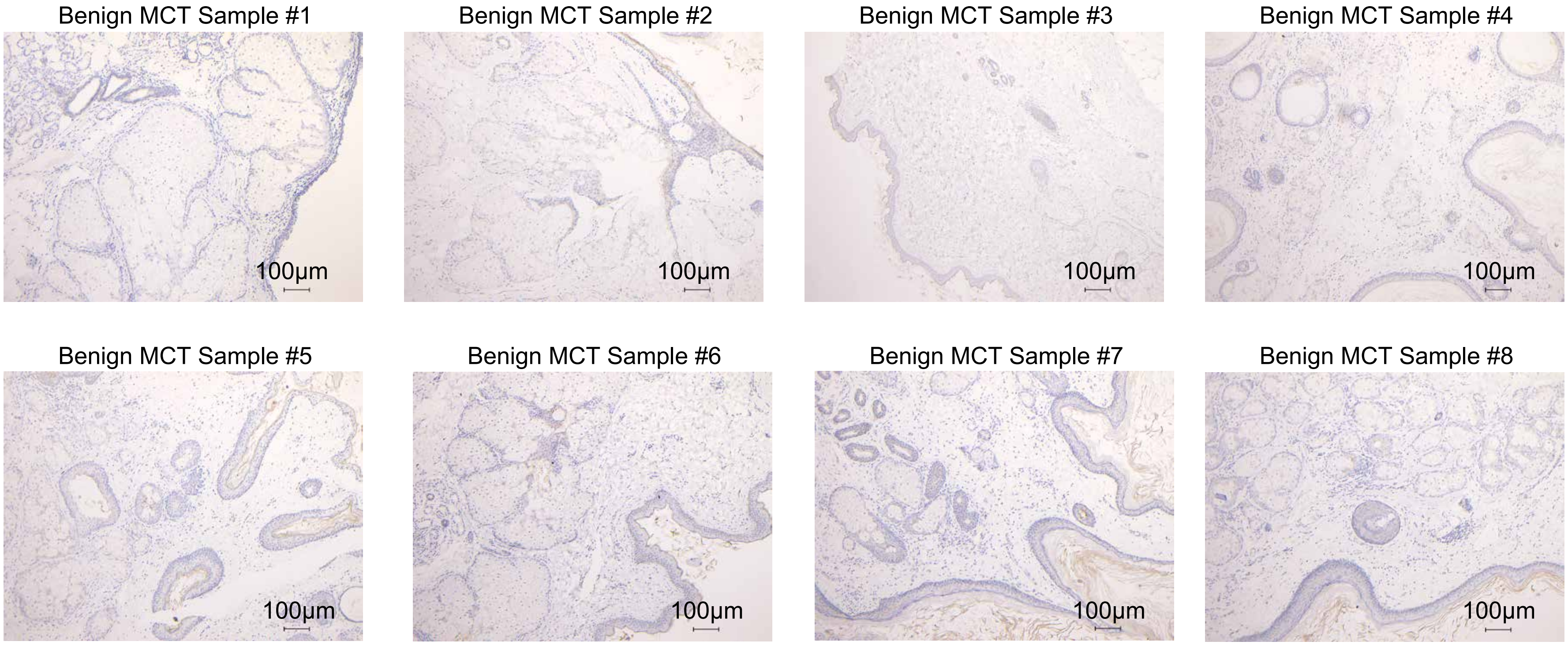

Supplementary Figure 8. No expression of XCL1 in mature cystic teratoma of the ovary

Immunohistochemical staining for XCL1 in 8 benign MCTs (100X magnification).

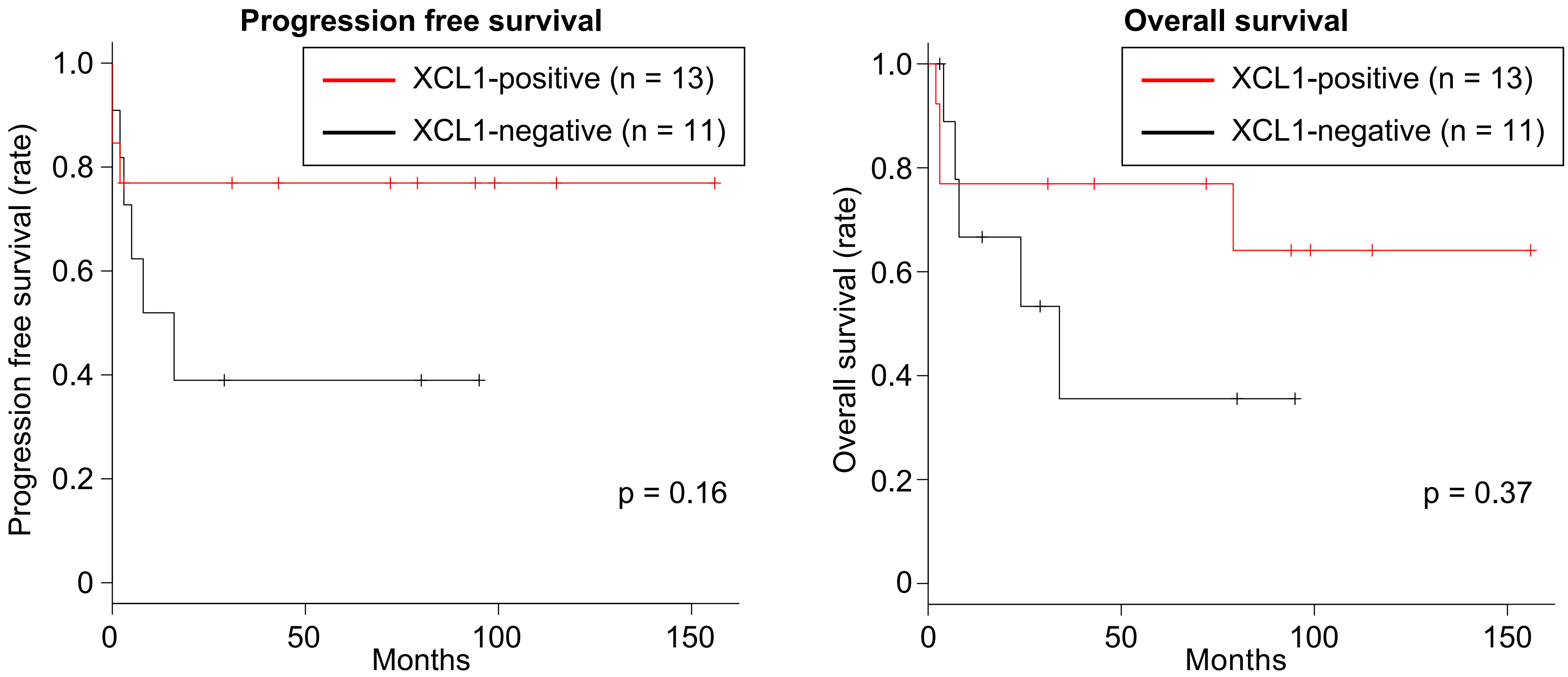

Supplementary Figure 9. The prognostic value of XCL1 *i*n 24 MCT-SCC/ASCs

Kaplan-Meier estimates of progression-free survival and overall survival for XCL1-positive and XCL1-negative patients.

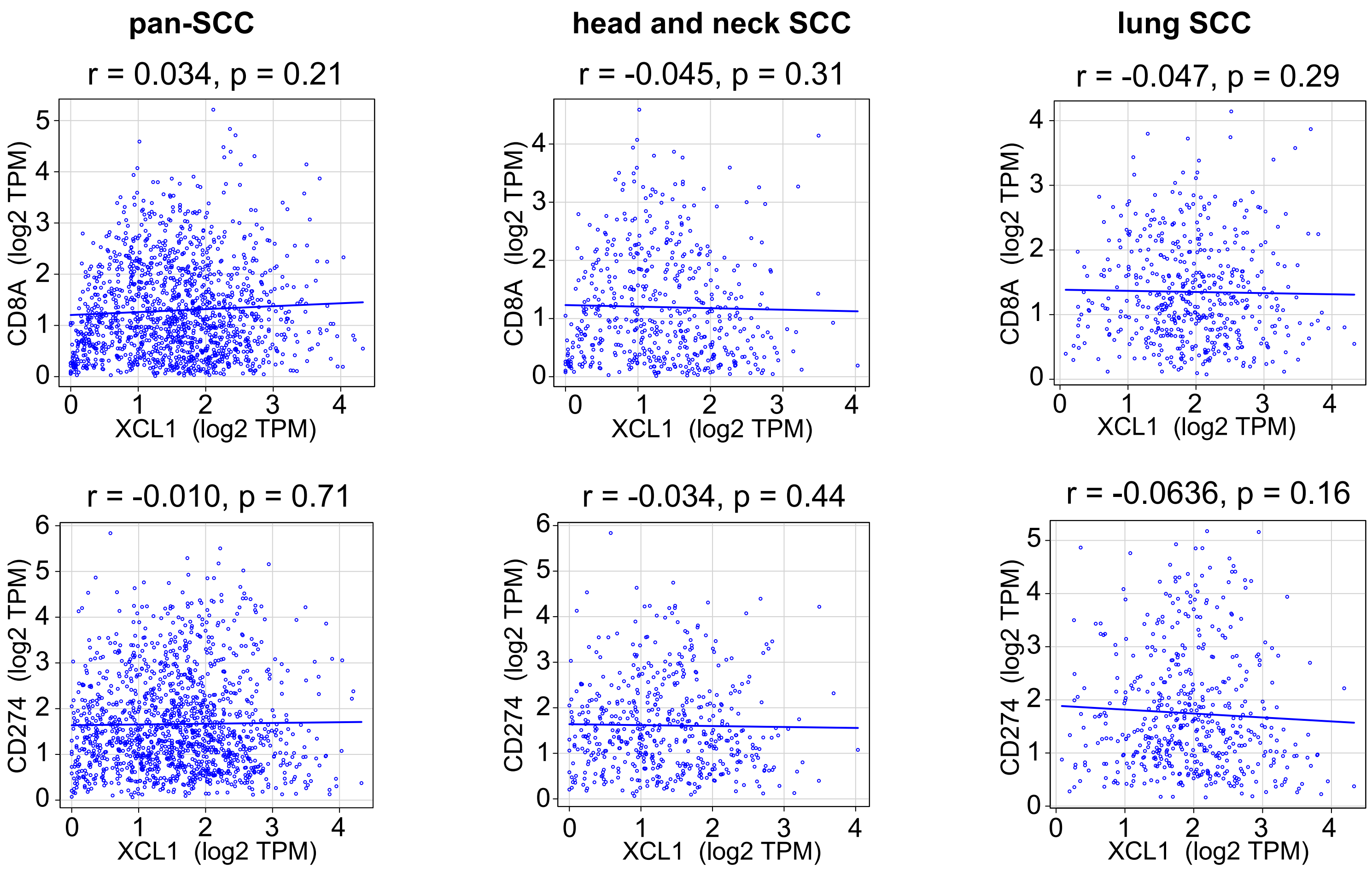

pan-SCC
head and neck SCC
lung SCC
r = 0.034, p = 0.21
r = -0.045, p = 0.31
r = -0.047, p = 0.29
r = -0.010, p = 0.71
r = -0.034, p = 0.44
r = -0.0636, p = 0.16
CD8A (log2 TPM)
CD274 (log2 TPM)
XCL1 (log2 TPM)

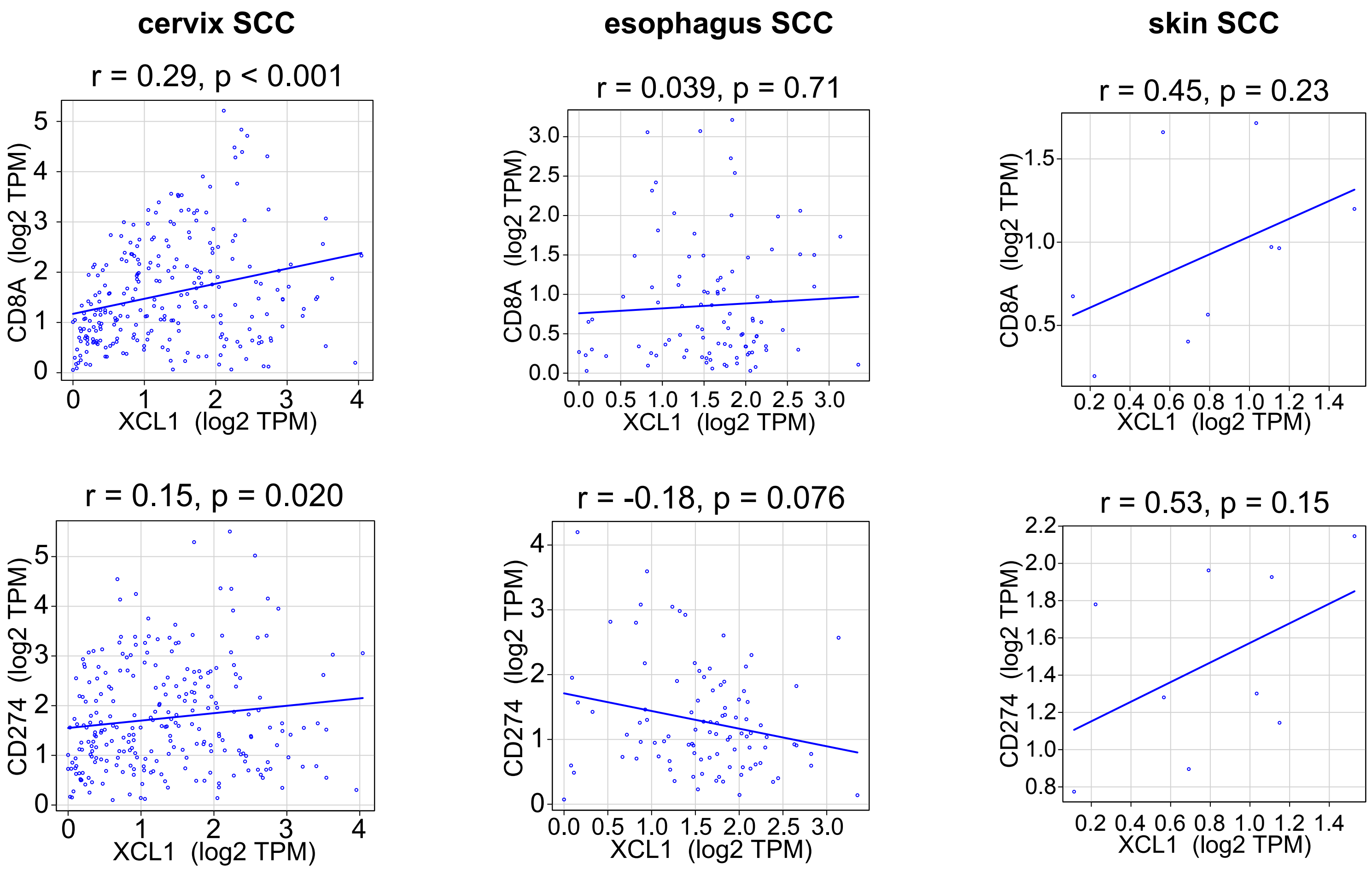

Supplementary Figure 10. The association between *XCL1* expression and *CD8A*/*CD274* expression in SCCs. The association between *XCL1* and *CD8A* (upper) or *CD274* (lower) mRNA expression in pan-SCCs and different SCCs is shown. Spearman rank correlation coefficient (r) and p -value are calculated by using Spearman's rank test.

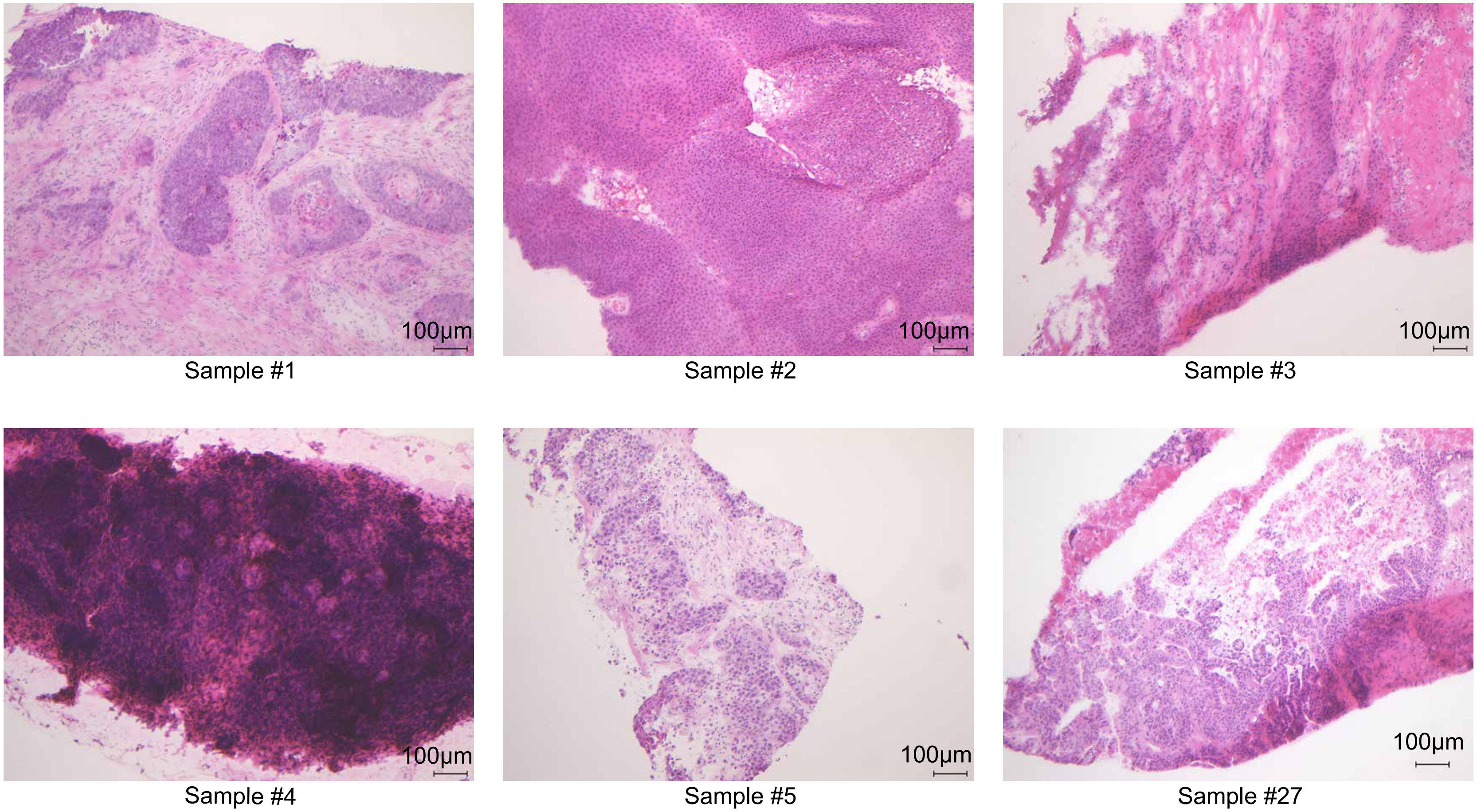

Supplementary Figure 11. Histological assessment of the tumor cell content in 6 fresh-frozen samples

Representative images of H&E stained 6 fresh-frozen samples of carcinomas arising from mature cystic teratoma of the ovary (100X magnification.)

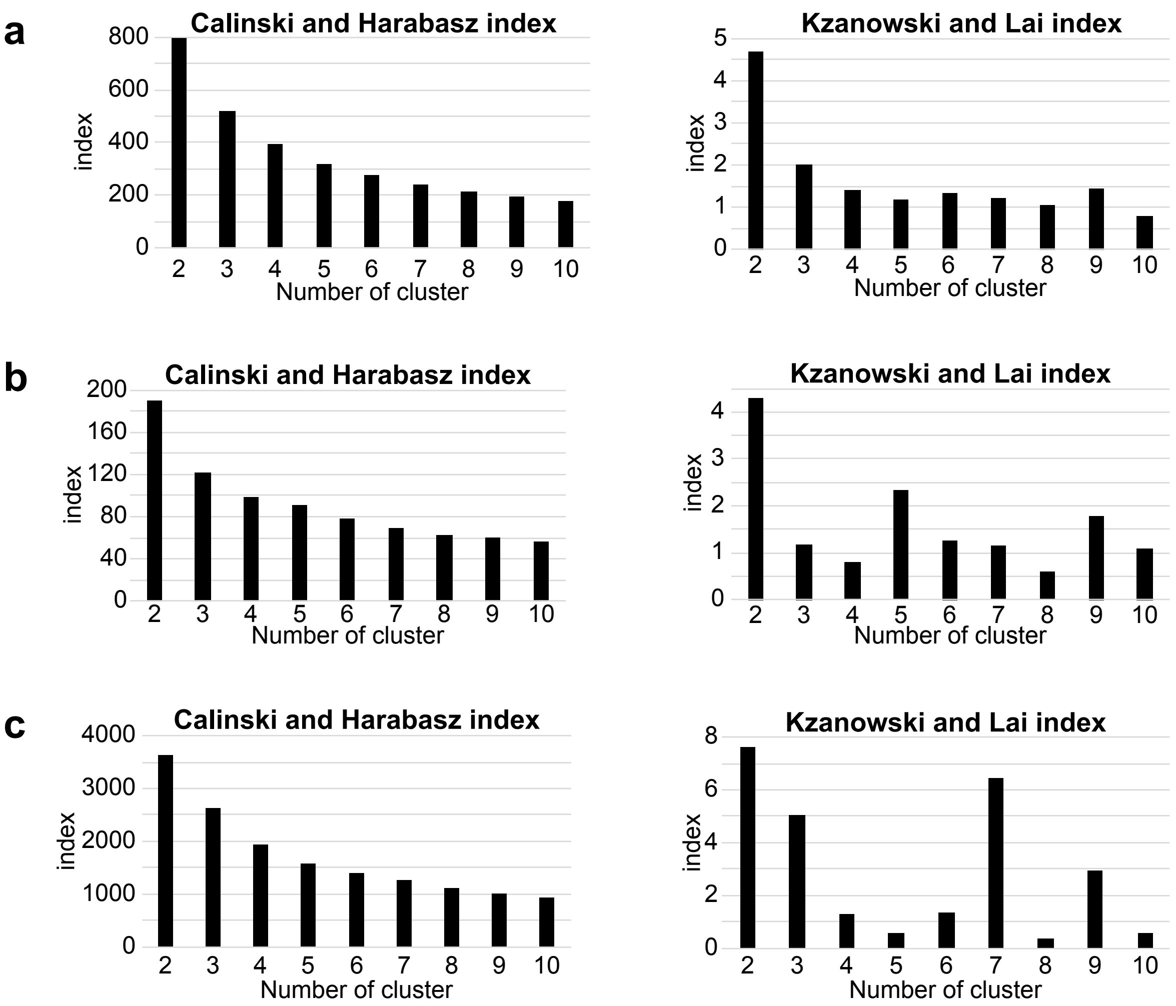

Supplementary Figure 12. A validity of clustering methods

The bar graph shows the Calinski and Harabasz index and the Kzanowski and Lai index of A (Fig. 2a), B (Fig. 3a) and C (Supplementary Fig. 6), respectively.

Gene	Sample	Chr.	Position	Amino acid substitution (coding DNA substitution)	Exome sequencing	RNA sequencing
					No. of reads containing mutation / total reads at mutation position (%)	
TP53	#1	17	7578196	V218G (T653G)	41/57 (72)	296/319 (93)
TP53	#3	17	7577085	E285K (G853A)	31/74 (42)	69/89 (79)
TP53	#4	17	7578199	V217E (T650A)	43/51 (84)	13/20 (65)
TP53	#5	17	7577568	C238Y (G713G)	37/54 (69)	139/139 (100)
PIK3CA	#1	3	178936989	Y557C (A1670G)	7/131 (5)	0/74 (0)
PIK3CA	#2	3	178936082	E542K (G1624A)	13/27 (48)	27/46 (46)
PIK3CA	#3	3	178936082	E542K (G1624A)	14/29 (48)	43/69 (62)
PIK3CA	#5	3	178936082	E542K (G1624A)	6/61 (10)	17/42 (40)

Supplementary Table 1. Validation of *TP53* and *PIK3CA* somatic mutations by RNA sequencing data

Gene	Sample	Chr	Position	Accession number	Amino acid substitution (coding DNA substitution)	Exome sequencing	RNA sequencing	OncoKB annotation	COSMIC	FATHMM
						No. of reads containing mutation / total reads at mutation position (%)				
TP53	#1	chr17	7578196	NM_000546	V218G (T653G)	41/57 (72)	296/319 (93)	likely oncogenic	Yes	0.99
TP53	#3	chr17	7577085	NM_000546	E285K (G853A)	31/74 (42)	69/89 (78)	likely oncogenic	Yes	0.99
TP53	#4	chr17	7578199	NM_000546	V217E (T650A)	43/51 (84)	13/20 (65)	likely oncogenic	Yes	0.84
TP53	#5	chr17	7577568	NM_000546	C238Y (G713A)	37/54 (69)	139/139 (100)	likely oncogenic	Yes	0.99
PIK3CA	#2	chr3	178936082	NM_006218	E542K (G1624A)	13/27 (48)	27/46 (59)	oncogenic	Yes	0.98
PIK3CA	#3	chr3	178936082	NM_006218	E542K (G1624A)	14/29 (48)	43/69 (62)	oncogenic	Yes	0.98
PIK3CA	#5	chr3	178936082	NM_006218	E542K (G1624A)	6/61 (10)	17/42 (40)	oncogenic	Yes	0.98
SETD2	#1	chr3	47098643	NM_014159	G2211X (G6631T)	65/74 (88)	23/31 (74)	likely oncogenic	NA	0.98
SETD2	#5	chr3	47142964	NM_014159	Q1667X (C4999T)	17/52 (33)	15/34 (44)	likely oncogenic	Yes	0.98
KDM6A	#2	chrX	44879855	NM_001291418	W148X (G444A)	18/58 (31)	18/63 (29)	likely oncogenic	NA	0.99
KMT2D	#3	chr12	49432359	NM_003482	S2927X (C8780G)	23/119 (19)	41/98 (42)	likely oncogenic	Yes	0.95
EP300	#3	chr22	41521980	NM_001429	S281X (C842G)	34/107 (32)	22/51 (43)	likely oncogenic	Yes	0.99
RB1	#1	chr13	49030478	NM_000321	Y651fs (1954dupA)	96/119 (81)	24/35 (69)	likely oncogenic	Yes	NA
MYC	#5	chr8	128750945	NM_002467	S161L (C482T)	190/372 (51)	284/428 (66)	predicted oncogenic	Yes	0.97
FBXW7	#3	chr4	153247289	NM_001013415	R387G (C1159G)	66/143 (46)	34/53 (64)	likely oncogenic	Yes	0.97
STK11	#5	chr19	1220629	NM_000455	S216Y (C647A)	7/18 (39)	38/40 (95)	likely oncogenic	NA	0.96
RUNX1	#2	chr21	36206728	NM_001122607	Q235X (C703T)	43/107 (40)	12/76 (16)	likely oncogenic	Yes	0.95
MGA	#2	chr15	41991137	NM_001080541	S697X (C2090G)	10/19 (53)	27/60 (45)	likely oncogenic	NA	0.97
MGA	#2	chr15	42052559	NM_001080541	W2201X (G6603A)	33/79 (42)	58/102 (57)	likely oncogenic	NA	0.93
MGA	#2	chr15	42052558	NM_001080541	W2201X (G6602A)	32/78 (41)	58/102 (57)	likely oncogenic	NA	0.93
FLT3	#3	chr13	28597546	NM_004119	D787Y (G2359T)	26/37 (70)	2/2 (100)	likely oncogenic	NA	1.00
DNMT3B	#2	chr20	31379488	NM_175850	R311X (C931T)	54/103 (52)	8/9 (89)	likely oncogenic	Yes	0.94
ZFHX3	#2	chr16	72821959	NM_006885	G3406fs (10215dupC)	54/107 (50)	2/16 (13)	likely oncogenic	Yes	NA
CASP8	#2	chr2	202131498	NM_033358	Q97X (C289T)	17/50 (34)	24/61 (39)	likely oncogenic	NA	0.99
ETV6	#3	chr12	12037511	NM_001987	G381E (G1142A)	26/86 (30)	52/145 (36)	predicted oncogenic	Yes	0.99
ALK	#1	chr2	29519851	NM_004304	G574R (G1720A)	119/320 (37)	0/0 (0)	not annotated	Yes	0.98
KLF5	#3	chr13	73636032	NM_001730	Q99E (C295G)	8/81 (10)	0/408 (0)	not annotated	Yes	0.97
KEAP1	#3	chr19	10600378	NM_012289	E493Q (G1477C)	13/60 (22)	168/373 (45)	not annotated	Yes	0.99
FGF3	#3	chr11	69625228	NM_005247	E189K (G565A)	12/95 (13)	0/0 (0)	not annotated	Yes	0.99
RPS6KA4	#3	chr11	64128609	NM_001300802	G156S (G466A)	46/58 (79)	136/142 (96)	not annotated	Yes	0.99
ERC1	#2	chr12	1137275	NM_001301248	S69C (C206G)	65/151 (43)	30/56 (54)	not annotated	Yes	0.99
GPHN	#5	chr14	67390968	NM_020806	P263S (C787T)	63/246 (26)	18/35 (51)	not annotated	Yes	0.99
HNRNPA2B1	#4	chr7	26236976	NM_031243	D87H (G259C)	31/130 (24)	188/1229 (15)	not annotated	Yes	0.98
MLLT4	#2	chr6	168352586	NM_001207008	L1494V (C4480G)	98/177 (55)	71/145 (49)	not annotated	Yes	0.98
HIST1H1E	#5	chr6	26156843	NM_005321	K75N (G225C)	23/57 (40)	11/11 (100)	not annotated	Yes	0.93

Chr = Chromosome, NA = Not Available

Supplementary Table 2. Pathogenic gene mutations identified by using whole-exome sequencing in five MCT-SCCs

Amplification

Gene	sample	copy number
AGO2	#4	8
MYC	#4	8
RICTOR	#4	7
JUN	#4	6
TERT	#4	6
MET	#4	6
FGF19	#5	7
FGF4	#5	7
FGF3	#5	7

Homozygous deletion

Gene	sample	copy number
CDKN2A	#4	0
CDKN2B	#4	0

Supplementary Table 3. Copy number amplification and homozygous deletion identified by using whole-exome sequencing in five MCT-SCCs

Sample	5' -Gene	3' -Gene	Chromosome 5' -Gene	Chromosome 3' -Gene	Breakpoint position 5' -Gene	Breakpoint position 3' -Gene	PRADA Discordant read (n)	PRADA Junction spanning read (n)	FusionCather Spanning pairs (n)	FusionCatcher Spanning unique reads (n)
#1	*TRIP12*	*FN1*	2	2	230693909	216253024	48	44	50	20
#2	*FGFR3*	*TACC3*	4	4	1808661	1741429	81	187	302	47
#4	*TNPO2*	*GADD45GIP1*	19	19	12825635	13065340	17	16	27	14

Supplementary Table 4. In-frame fusion transcripts identified in 5 MCT-SCCs by using both PRADA and FusionCatcher

Gene	Sample	Chr	Position	Accession number	Coding DNA substitution	Amino acid substitution	Mutant Allele Frequency (MAF)
TP53	#6	chr17	7576885	NM_001126112	960delG	K321fs	0.20
TP53	#7	chr17	7578382	NM_001126112	C548G	S183X	0.42
TP53	#8	chr17	7578380	NM_001126112	G550C	D184H	0.73
TP53	#8	chr17	7578513	NM_001126112	G417C	K139N	0.71
PIK3CA	#6	chr3	178936091	NM_006218	G1633A	E545K	0.13
TERT	#6	chr5	1295250	NM_198253	C-146T promoter variant	NA	0.11
BRAF	#7	chr7	140453155	NM_004333	G1780C	D594H	0.38
CDKN2A	#7	chr9	21971036	NM_000077	G322T	D108Y	0.50
AKT1	#7	chr14	105246551	NM_001014432	G49A	E17K	0.30
CTNNB1	#8	chr3	41266113	NM_001904	C110G	S37C	0.67
RB1	#8	chr13	48947575	NM_000321	1166dupT	L389fs	0.81
RAD51	#8	chr15	41011016	NM_001164269	G452A	R151Q	0.11

Gene	Sample	Chr	Copy number alteration
CDKN2A	#7	chr9	loss
CDKN2B	#7	chr9	loss
PTEN	#8	chr10	loss
CDKN1B	#8	chr12	loss
SUFU	#8	chr10	loss
SMAD4	#8	chr18	loss

Chr = Chromosome, NA = Not Available

Supplementary Table 5. Curated pathogenic alterations identified by using target sequencing in three MCT-SCCs

Gene	Oncogenic Alteration	Cancer type	Drug	Highest level of evidence	Sample
PIK3CA	E542K / E545K	Breast Cancer	PI3K inhibitor	1	#2<br>#3<br>#5<br>#6
FGFR3-TACC3	*FGFR3* fusion	Breast Cancer<br>All solid tumor	FGFR inhibitor	1	#2
FLT3	D787Y (G2359T)	Acute Myeloid Leukemia	Multi kinase inhibitor	1	#3
MET	Amplification	Non-Small Cell Lung Cancer<br>Renal Cell Carcinoma	Multi kinase inhibitor	2	#4
AKT1	E17K	Breast Cancer<br>Endometrial Cancer<br>Ovarian cancer	Akt inhibitor	3	#7
BRAF	D594H	Histiocytosis	MEK inhibitor	3	#7
CDKN2A	loss / D108Y	All Solid Tumors	CDK4/6 inhibitor	4	#7
PTEN	loss	All Solid Tumors	PI3K inhibitor	4	#8
KDM6A	W148X (G444A)	All Solid Tumors	EZH2 inhibitor	4	#2

Supplementary Table 6. Details of druggable oncogenic alterations identified in 8 MCT-SCCs

Sample	Sequencing platform	Tumor mutation burden (counts/Mb)
#1	Whole exome Sequencing	1.2
#2	Whole exome Sequencing	9.9
#3	Whole exome Sequencing	6.6
#4	Whole exome Sequencing	1.5
#5	Whole exome Sequencing	2.3
#6	Target Sequencing	0.8
#7	Target Sequencing	6.2
#8	Target Sequencing	7.7

Supplementary Table 7. Tumor mutation burden in 8 MCT-SCCs

	XCL1-positive (n = 13)	XCL1-negative (n = 11)	p-value
**Median age (range)**	59.5 (36-73)	60 (33-79)	p = 0.44
**stage**			
Early (stage I)	8	4	p = 0.41
Advanced (stage II-IV)	5	7	
**Histology**			
SCC	11	10	p = 1.00
ASC	2	1	
**Classification**			
keratinizing	4	9	p = 0.019
nonkeratinizing	9	2	

Supplementary Table 8. Comparison of clinicopathological findings between XCL1-positive and XCL1-negative patients

Survival	Characteristics	Multi-variate analysis	
		HR (95% CI)	P-value
Progression free survival	CD8 high (vs. low)	0.085 (0.0080-0.89)	0.04
	XCL1 positive (vs. negative)	0.88 (0.0087-9.0)	0.92
	PD-L1 positive (vs. negative)	3.4 (0.32-36)	0.31
Overall survival	CD8 high (vs. low)	0.047 (0.0033-1.3)	0038
	XCL1 positive (vs. negative)	2.2 (0.13-37)	0.58
	PD-L1 positive (vs.negative)	3.1 (0.38-24)	0.29

Supplementary Table 9. Multi-variate analysis by using XCL1, CD8 and PD-L1 expression in 24 MCT-SCC/ASCs

Target	Forward primer	Reverse primer
XCL1	AAGAGGACCTGTGTGAGCCT	GGCTTGTGGATCAGCACAGA
ACTB	TGGCACCCAGCACAATGAA	CTAAGTCATAGTCCGCCTAGAAGCA

Supplementary Table 10. Details of the primers used in this study

Supplementary Data 1. Significantly overrepresented pathways in cluster 1 compared with cluster 2 using GO gene sets and HALLMARK gene sets

Supplementary Data 2. Significantly overrepresented pathways in cluster L compared with cluster R using GO gene sets and HALLMARK gene sets
